# Comparative Genomics of Cyanobacterial Symbionts Reveals Distinct, Specialized Metabolism in Tropical *Dysideidae* Sponges

**DOI:** 10.1128/mBio.00821-19

**Published:** 2019-05-14

**Authors:** Michelle A. Schorn, Peter A. Jordan, Sheila Podell, Jessica M. Blanton, Vinayak Agarwal, Jason S. Biggs, Eric E. Allen, Bradley S. Moore

**Affiliations:** a Center for Marine Biotechnology and Biomedicine, Scripps Institution of Oceanography, University of California, San Diego, California, USA; b School of Chemistry and Biochemistry, School of Biological Sciences, Georgia Institute of Technology, Atlanta, Georgia, USA; c University of Guam Marine Laboratory, UoG Station, Mangilao, Guam, USA; d Center for Microbiome Innovation, University of California, San Diego, California, USA; e Division of Biological Sciences, University of California, San Diego, California, USA; f Skaggs School of Pharmacy and Pharmaceutical Sciences, University of California, San Diego, California, USA; University of Hawaii at Manoa; Princeton University; Stony Brook University

**Keywords:** biosynthesis, cyanobacteria, halogenated compounds, marine microbiology, metagenomics, natural products, nonribosomal peptide synthetase

## Abstract

Marine sponges are recognized as valuable sources of bioactive metabolites and renowned as petri dishes of the sea, providing specialized niches for many symbiotic microorganisms. Sponges of the family *Dysideidae* are well documented to be chemically talented, often containing high levels of polyhalogenated compounds, terpenoids, peptides, and other classes of bioactive small molecules. This group of tropical sponges hosts a high abundance of an uncultured filamentous cyanobacterium, Hormoscilla spongeliae. Here, we report the comparative genomic analyses of two phylogenetically distinct *Hormoscilla* populations, which reveal shared deficiencies in essential pathways, hinting at possible reasons for their uncultivable status, as well as differing biosynthetic machinery for the production of specialized metabolites. One symbiont population contains clustered genes for expanded polybrominated diphenylether (PBDE) biosynthesis, while the other instead harbors a unique gene cluster for the biosynthesis of the dysinosin nonribosomal peptides. The hybrid sequencing and assembly approach utilized here allows, for the first time, a comprehensive look into the genomes of these elusive sponge symbionts.

## Abstract (precis)

Natural products provide the inspiration for most clinical drugs. With the rise in antibiotic resistance, it is imperative to discover new sources of chemical diversity. Bacteria living in symbiosis with marine invertebrates have emerged as an untapped source of natural chemistry. While symbiotic bacteria are often recalcitrant to growth in the lab, advances in metagenomic sequencing and assembly now make it possible to access their genetic blueprint. A cell enrichment procedure, combined with a hybrid sequencing and assembly approach, enabled detailed genomic analysis of uncultivated cyanobacterial symbiont populations in two chemically rich tropical marine sponges. These population genomes reveal a wealth of secondary metabolism potential as well as possible reasons for historical difficulties in their cultivation.

## INTRODUCTION

Eons of natural selection have produced exquisite, naturally derived chemical diversity that humans have exploited for medicinal potential for centuries, including the majority of approved pharmaceutical drugs (1). Marine invertebrates, particularly sponges, have emerged as highly rewarding reservoirs of natural chemical diversity (2). These ancient, sessile animals are unable to physically evade predators, and it is thought that many have formed symbioses with microbes that can produce chemical defenses while benefiting from a nutrient-rich environment (3). Access to these symbionts and their full chemical repertoires has been limited due to difficulties in culturing many symbiotic microbes. The genomic revolution has provided new tools for sustainably interrogating complex metagenomes and has exposed the surprising breadth of biosynthetic capabilities of talented natural-product-generating microbes (4).

**FIG 1:**
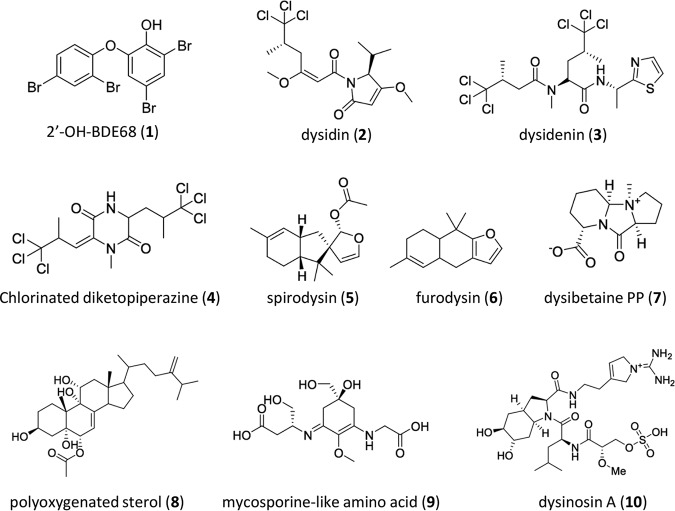
Representative secondary metabolites previously isolated from *Lamellodysidea herbacea* specimens.

The *Lamellodysidea* (formerly *Dysidea*) genus within the *Dysideidae* family is particularly prolific in its variety of bioactive natural products (Fig. 1). This family’s best-known class of compounds, due to the environmental toxicity of their anthropogenic counterparts, is the polybrominated diphenyl ethers (PBDEs), e.g., compound 1, first isolated in 1972 from Dysidea herbacea and subsequently from numerous *Dysideidae* sponges (5, 6). Astonishingly, PBDEs can make up over 10% of the sponge’s dry weight, with cell sorting (7) and microbiome sequencing studies (8) attributing these abundant molecules to the dominant cyanobacterial symbiont. Numerous other distinct structural classes of molecules have been isolated from *Lamellodysidea* specimens (Fig. 1), including polychlorinated peptidic molecules (e.g., compounds 2 to 4) (9, 10). Some of these chlorinated metabolites have been credited to the dominant cyanobacterial symbiont rather than the sponge itself (11–13). On the other hand, several distinct sesquiterpene molecules (e.g., compounds 5 and 6) that colocalize with sponge cells rather than cyanobacteria (12) have also been characterized from *Lamellodysidea* (14). At least four other unique classes of metabolites (e.g., compounds 7 to 10) have been isolated from Lamellodysidea herbacea with no experimental evidence of the true producer within the sponge holobiont (15–19).

Such a wealth of chemistry from one family of sponges, with a focus on one species here, *Lamellodysidea herbacea*, is remarkable, but with the abundance of microbial biodiversity residing in sponges, the true source of these diverse compounds is difficult to decipher. In some cases, sequencing has shown that the dominant symbiont of an invertebrate assemblage contains the biosynthetic machinery to be a major source of natural products (20). Genomic sequencing has shown this to be the case for the marine ascidian Lissoclinum patella and its uncultured cyanobacterial symbiont, Prochloron didemni, and the marine sponge Theonella swinhoei and its uncultured bacterial symbiont, Entotheonella swinhoei (21, 22).

A defining feature of *Lamellodysidea* sponges is the persistent presence of a filamentous cyanobacterial symbiont, Hormoscilla spongeliae (formerly Oscillatoria spongeliae) (23), with distinct strains inhabiting morphologically discrete hosts (24, 25). Despite repeated attempts, these symbionts have been recalcitrant to culturing efforts and are therefore assumed to be obligate symbionts, unable to live outside their host system (26). Likewise, field studies have shown that when cyanobacterial photosynthesis is inhibited by shading, *Lamellodysidea* sponges have a higher mortality rate (27), suggesting that the host sponge critically depends on symbiont carbon fixation, making this a bidirectional obligate symbiosis.

Recently, we reported the biosynthetic gene clusters (BGCs) responsible for PBDE synthesis within the genomes of three *Dysideidae* cyanobacterial symbionts, establishing bacterial origins for at least one major metabolite isolated from *L. herbacea* sponges (8). However, origins of the majority of secondary metabolites isolated from *L. herbacea* specimens have not yet been addressed, and the underlying causes of our inability to cultivate many sponge symbionts are still unknown. Obtaining high-quality draft genomes of the uncultured cyanobacterial symbionts has allowed us to investigate both of these questions, providing potential reasons for their seemingly obligate symbiont lifestyle and revealing differing biosynthetic capacities for producing secondary metabolites of varied chemotypes.

## RESULTS AND DISCUSSION

### Enriched MAGs.

Two *L. herbacea* sponges, named GUM007 and GUM202, exhibited different chemotypes: GUM007 contained no PBDEs, while GUM202 contained an abundance of PBDEs. Differences in secondary metabolite biosynthetic potential in the two sponge symbionts were explored using a hybrid sequencing approach to generate metagenome-assembled genomes (MAGs). Short-read metagenomic sequencing alone often fails to produce sufficiently contiguous sequences for examining long, multigene biosynthetic gene clusters (BGCs) (28). Recently developed technologies, such as Pacific Biosciences (PacBio) single-molecule real-time sequencing, enable the high-throughput generation of very long reads. Reduced accuracy of these long reads can be offset by supplementation with high-accuracy, short reads obtained from Illumina sequencing. This strategy was previously shown to improve assembly quality of over 30 binned genomes from a deeply sequenced sponge microbiome (29). To enhance the purity of DNA from yet-uncultivable *Hormoscilla* symbionts, we isolated cyanobacterial trichomes from the less dense sponge cells by pressing the sponge tissue to exude cyanobacterial cells and separating them by centrifugal partitioning (see Fig. S1 in the supplemental material) (26).

10.1128/mBio.00821-19.2 FIG S1 Workflow for processing whole sponges and enriched cyanobacteria fractions. Simplified workflow showing parallel processing and sequencing for whole-sponge metagenomes using Illumina and enriched cyanobacteria using PacBio. Two different hybrid assembly methods were used to obtain genome assemblies. Download FIG S1, PDF file, 0.3 MB. Copyright © 2019 Schorn et al. 2019 Schorn et al. This content is distributed under the terms of the Creative Commons Attribution 4.0 International license.

Hybrid assemblies using phylogenetically classified Illumina and PacBio reads resulted in high-quality draft MAGs when assessed according to published standard recommendations promulgated by the Genomics Standards Consortium (Table 1) (30). The GUM007_hs genome consisted of 64 scaffolds, with an average length of 97 kb and 94.46% completeness, according to CheckM (31). GUM202_hs consisted of 70 scaffolds, with an average length of 98 kb and 97.41% completeness. These two population genomes have an average nucleotide identity (ANI) of 96.18%, an average amino acid identity (AAI) of 93.12%, and a 16S rRNA nucleotide pairwise identity of 97.9%. Recent standards for classifying uncultivated microbes indicate that the evolutionary distance of these two populations is on the threshold between genus and species, with two out of three criteria indicating that they are different species (32). While these two populations are phylogenetically closely related, their overall genome organization contains only limited regions of synteny (Fig. S2). This biological strategy of retaining closely related conserved genes, while allowing greater genomic plasticity in other regions, such as genomic islands where secondary metabolism genes reside, has been reported for other chemically talented bacteria (33).

**TABLE 1:** Assembly and quality statistics for GUM_hs genomes^*a*^

Statistic	Genome	
	GUM007_hs	GUM202_hs
Analysis project type	MAG	MAG
Taxon ID	Multimarker	Multimarker
Assembly software	IDBA-UD, Celera, SSPACE-LongRead	IDBA-UD, hybridSPAdes
Assembly quality	High-quality draft	High-quality draft
rRNA genes	23S, 16S, 5S	23S, 16S, 5S
CheckM completeness (%)	94.46	97.41
CheckM contamination	2.24	2.00
No. of scaffolds	64	70
Avg length of scaffolds (bp)	97,372	98,137
Longest scaffold (bp)	345,613	304,315
Estimated genome size (Mb)	6.2	6.8
*N*_50_ (bp)	169,688	161,164
% GC	47.8	47.5
Bin parameters	% GC, nucleotide composition, coverage, and taxonomic assignment	% GC, nucleotide composition, coverage, and taxonomic assignment
Binning software	Custom/manual	Custom/manual

a General assembly and quality statistics for the *Hormoscilla* metagenome-assembled genomes (MAGs) from GUM007 and GUM202. Both MAGs meet the MIMAG standards for high-quality draft MAGs. Two-way ANI was 96.18%, two-way AAI was 93.12%, and 16S rRNA nucleotide pairwise identity was 97.9%.

10.1128/mBio.00821-19.3 FIG S2 MUMmer plot showing synteny between GUM007_hs and GUM202_hs. Overall synteny of the two symbiont genomes, with purple dots representing forward regions of the genome that align and blue dots representing reverse regions of the genomes that align. Download FIG S2, PDF file, 0.3 MB. Copyright © 2019 Schorn et al. 2019 Schorn et al. This content is distributed under the terms of the Creative Commons Attribution 4.0 International license.

**FIG 2:**
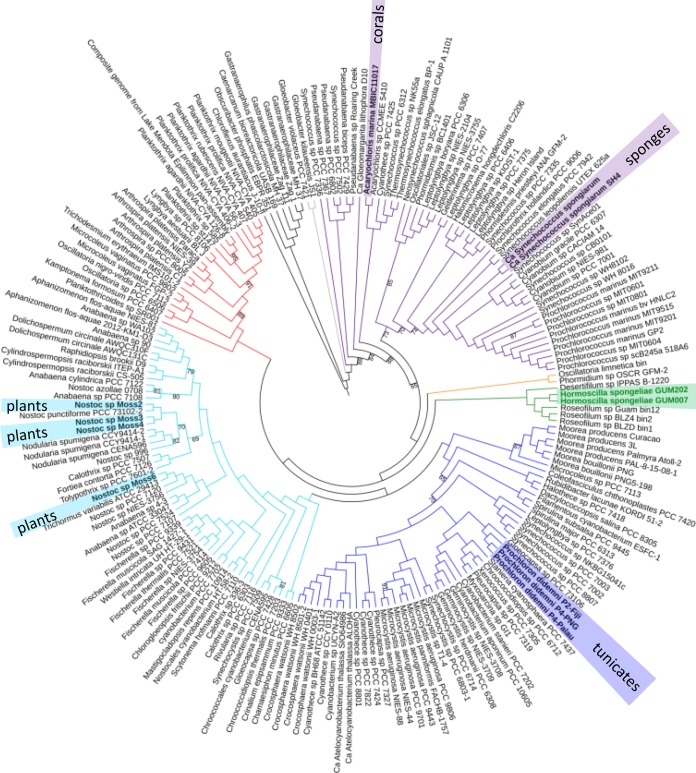
Multilocus sequence analysis of diverse cyanobacteria. One hundred ninety-seven cyanobacterial genomes containing 25 housekeeping genes in single copy were used to construct a maximum-likelihood tree. The two *Hormoscilla* spp. are highlighted in green, and their nearest sister clade is the *Roseofilum* spp., known coral pathogens. Other close relatives, *Desertifilum* sp., *Phormidium* sp., and Oscillatoria limnetica, have been found growing in microbial mats. Other symbiotic cyanobacteria are highlighted throughout the tree. Only bootstrap values between 65 and 95 are shown; all other bootstrap values are above 95.

A well-supported multilocus sequence analysis phylogenetic tree gives a phylogenetic reference for how these unusual symbionts are related to other cyanobacterial taxa (Fig. 2). The two *Hormoscilla* MAGs clade together and, within the realm of sequenced organisms, are most closely related to multiple *Roseofilum* MAGs. *Roseofilum* spp. are filamentous cyanobacteria found in microbial assemblages that afflict corals with a disease called black band disease (BBD) (34). Comparative genomics of five *Roseofilum* spp., which have never been found living independently, have shown that they are reliant on other members of the microbial consortium, as many filamentous cyanobacteria are, and as *Hormoscilla* appears to be (34). The deep-branching clade that contains both *Hormoscilla* and *Roseofilum* spp. is sparsely populated, perhaps due to their participation in microbial consortia, thus making them difficult to culture.

### Genomic hallmarks of a symbiotic lifestyle.

*Hormoscilla* symbionts were initially expected to contain a pared-down genome with loss of metabolic functions, such as those seen in “*Candidatus* Synechococcus spongiarum,” yet-uncultured cyanobacterial symbionts of diverse sponges (35, 36). However, estimated sizes of the *Hormoscilla* genomes ranged between 6.2 and 6.8 Mb, comparable to other free-living filamentous cyanobacteria. The *Hormoscilla* genomes did share some other symbiont specific characteristics of “*Ca.* Synechococcus spongiarum” genomes. Ankyrin repeat proteins (COG0666) and leucine-rich repeat (LRR) proteins (COG4886) are present in both *Hormoscilla* genomes and are postulated to play a role in bacterium-sponge recognition. “*Ca*. Synechococcus spongiarum” genomes exhibited a loss of low-molecular-weight peptides involved in photosystem II, and PsbJ was lost in both *Hormoscilla* and PsbX in GUM007_hs. Both *Hormoscilla* genomes were also missing several oxidative-stress-related genes, including glutathione peroxidase (EC 1.11.1.9), gamma-glutamyl transpeptidase (EC 2.3.2.2), and superoxide dismutase (SOD) (36).

The mounting evidence of uncultured symbionts from marine invertebrates may point to an alternative evolutionary phenomenon: retaining a large, chemically talented genome can impart evolutionary advantages to the holobiont (79). Seemingly obligate endosymbionts such as *Hormoscilla*, *P. didemni* (37), and *Entotheonella* spp. (38), which retain a large genome despite reliance on their host, may represent another evolutionary mechanism in which the maintenance of extended genomic repertoires sustains the symbiotic interaction. If this were the case, these symbionts might not complete the genome streamlining process because losing their biosynthetic capabilities to manufacture metabolically expensive chemicals would reduce the evolutionary fitness of the holobiont.

### Missing essential gene analysis.

To understand why *Hormoscilla* species cannot be cultured in the laboratory despite their lack of canonical symbiotic traits, we compared metabolic pathways and gene essentiality with an extensively characterized free-living cyanobacterium. A curated genome-scale model (GEM) has been developed to determine the genes essential to sustain life in a photosynthetic organism using the model cyanobacterium Synechococcus elongatus PCC 7942 (39). This GEM is informed not just by metabolic modeling data but also by genome-wide gene essentiality analysis, determined from ∼250,000 transposon mutants to assign genes as essential, beneficial, or nonessential, making it the only large-scale study of the genes essential to sustaining photosynthetic life (40). Although *S. elongatus* and *Hormoscilla* belong to different orders (*Synechococcales* and *Oscillatoriales*, respectively), and differences in gene content are expected, the comparison allowed us to leverage the extensive, experimentally validated data about gene essentiality in a free-living photosynthetic organism to expose missing essential genes in a cyanobacterial symbiont.

An initial comparison of the GUM_hs genes versus the *S. elongatus* GEM revealed a combined list of 155 genes missing in both GUM_hs genomes. Missing genes were then converted to corresponding KEGG Ontology (KO) (41) and pfam (42) models for a more robust search. Any models that remained unfound in the *Hormoscilla* MAGs were cross-referenced with gene essentiality data for *S. elongatus* PCC 7942, resulting in seven KOs/pfams that are missing in the GUM_hs assemblies yet essential for *S. elongatus* (see Table S1 in the supplemental material).

10.1128/mBio.00821-19.9 TABLE S1 Essential genes from Synechococcus elongatus PCC 7942 missing from GUM202_hs and GUM007_hs. The essential gene analysis using the well-curated *S. elongatus* PCC 7942 metabolic model as a reference, resulting in eight genes that are essential for *S. elongatus* and missing in both GUM007_hs and GUM202_hs. Subsequent inspection of each pathway showed that only the biosynthetic pathway for histidine was actually incomplete. Download Table S1, PDF file, 0.6 MB. Copyright © 2019 Schorn et al. 2019 Schorn et al. This content is distributed under the terms of the Creative Commons Attribution 4.0 International license.

Both population genomes predict that *Hormoscilla* are prototrophic for all amino acids except histidine. The missing essential gene analysis exposed a lack of imidazole glycerol phosphate dehydratase (HisB), the enzyme responsible for the sixth step in histidine biosynthesis, in both GUM_hs genomes (Fig. S3). No alternative pathway for histidine biosynthesis was found. Due to the phylogenetic distance of *S. elongatus*, a more closely related filamentous cyanobacterium with a complete genome sequence, Moorea producen*s*, was also used for comparison (43). A BLAST search using *hisB* from *Moorea producens* did not reveal any candidate orthologs in the GUM_hs genomes. Furthermore, *hisB* from *M. producens* and the pfam HMM for HisB (PF00475) were used to search the assembled metagenomes, and no significant hits to cyanobacteria were found. While it is possible that this gene was not assembled from the sequence data generated, it may also indicate that *Hormoscilla* species are histidine auxotrophs, requiring histidine supplementation from their hosts or other members of the microbiome. Alternatively, there could be a complementary gene encoding a novel dehydratase not yet discovered to replace HisB in histidine biosynthesis.

10.1128/mBio.00821-19.4 FIG S3 Histidine metabolism in *Hormoscilla* versus *S. elongatus* PCC 7942. This metabolic map was made using ec2kegg comparing histidine metabolism in *Hormoscilla* with that in *S. elongatus* PCC 7942. Yellow boxes indicate that the corresponding EC number is present in both reference and query genomes. Green boxes are EC numbers found only in the reference genome (*S. elongatus* PCC 7942). Red boxes are EC numbers present only in the query genome (GUM007_hs and GUM202_hs). Comparison of the biosynthetic pathway for the amino acid, histidine, in *S. elongatus*, GUM007_hs, and GUM202_hs shows an apparent lack of an essential enzyme, imidazole glycerol-phosphate dehydratase (EC 4.2.1.19, blue box), which performs the sixth step in histidine biosynthesis. Download FIG S3, PDF file, 0.03 MB. Copyright © 2019 Schorn et al. 2019 Schorn et al. This content is distributed under the terms of the Creative Commons Attribution 4.0 International license.

The remaining six genes that are essential for *S. elongatus* but missing in both GUM_hs assemblies were determined to have complementary pathways for completing the required reactions (Table S1 and Text S1). Additionally, we examined the ability of *Hormoscilla* symbionts to produce important cofactors. Both GUM_hs genomes are missing canonical genes encoding the enzymes performing the last step in thiamine biosynthesis, which are present in both *S. elongatus* and *M. producens* (Fig. S4a). This suggests that *Hormoscilla* symbionts may be unable to make thiamine. The pathway for production of biotin, an important cofactor in fatty acid and amino acid biosynthesis, is also incomplete in *Hormoscilla*, with key enzymes BioA and BioC missing (Fig. S4b). Interestingly, *M. producens* is also missing BioA, but the entire biotin pathway is present in a persistent, uncultured heterotrophic bacterium growing together with *M. producens* (44). *M. producens* does not grow axenically and always has a consortium of heterotrophic bacteria growing with it; thus, it is possible that neither *M. producens* nor *Hormoscilla* symbionts can make biotin on their own and rely on heterotrophic bacteria for provision of this important cofactor. Future cultivation efforts of *Hormoscilla* symbionts should include addition of histidine, thiamine, and biotin to address possible deficits in these biosynthetic pathways.

10.1128/mBio.00821-19.1 TEXT S1 Additional information, including the JGI genome IDs used in the multilocus sequence analysis, expanded missing essential gene analysis, and detailed chemical elucidation data for PBDEs and desoxydysinosins. Download Text S1, PDF file, 0.1 MB. Copyright © 2019 Schorn et al. 2019 Schorn et al. This content is distributed under the terms of the Creative Commons Attribution 4.0 International license.

10.1128/mBio.00821-19.5 FIG S4 Thiamine and biotin metabolism in *Hormoscilla* versus *S. elongatus* and *Moorea producens.* These metabolic maps were made using ec2kegg and follow the same coloring scheme. Yellow boxes indicate that the corresponding EC number is present in both reference and query genomes. Green boxes are EC numbers found only in the reference genome (*S. elongatus* PCC 7942 in panel a and *M. producens* in panel b). (a) Both GUM007_hs and GUM202_hs genomes appear to be lacking the three enzymes involved in the last step of thiamine biosynthesis (ECs 3.1.3.1, 3.1.3.2, and 3.1.3.100, red box). (b) The biotin pathway appears incomplete in both *M. producens* and *Hormoscilla*. Putative homologues of BioK, which can replace BioH in pimeloyl-CoA biosynthesis, were found in *M. producens* and *Hormoscilla*. However, no homologues of BioA (ECs 2.6.1.62 and 2.6.1.105, red box) have been found in either species. Download FIG S4, PDF file, 0.05 MB. Copyright © 2019 Schorn et al. 2019 Schorn et al. This content is distributed under the terms of the Creative Commons Attribution 4.0 International license.

### Distinct specialized metabolism of the GUM007 and GUM202 *Hormoscilla* symbionts.

**FIG 3:**
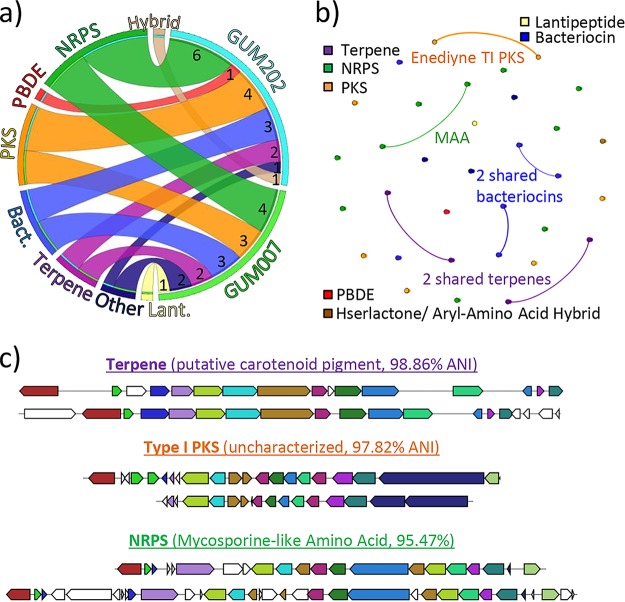
Secondary metabolite biosynthetic gene clusters (BGCs) of two *Hormoscilla* populations. An overview of the variety of BGCs found in the two *Hormoscilla* populations, including shared and distinct BGCs. (a) Number and classes of BGCs in the GUM_hs genomes. (b) Gene cluster similarity network where each node represents a gene cluster and those with similarity over a 0.4 threshold are connected by a line. The weight of the line indicates higher similarity for bolder lines. (c) MultiGeneBlast comparison of similar clusters displays gene synteny.

The near-completeness of the two genomes afforded us a comprehensive survey of identifiable BGCs within these *Hormoscilla* specimens. AntiSMASH 4.1.0 (45) results for both GUM007_hs and GUM202_hs are shown in Fig. 3a. A total of 15 BGCs (after splitting an unlikely nonribosomal peptide synthetase [NRPS]/bacteriocin hybrid cluster) were found in GUM007_hs, and 18 were found in GUM202_hs. A BGC similarity network, created using BiG-SCAPE (46), revealed that the genomes share six clusters, making two-thirds of the BGCs unique to each organism (Fig. 3b). Four out of the six shared clusters have ANI values above the whole-genome ANI score (96.18%), which may suggest that these gene clusters are conserved for the benefit of the symbiosis.

Of those clusters shared, half have predicted structures. Two terpene clusters, with ANIs of 90.24% and 98.86%, likely encode carotenoid pigments, due to the presence of lycopene synthases, and are not predicted to be implicated in the production of characteristic sesquiterpenes isolated from *L. herbacea* sponges. Notably, no sesquiterpene biosynthetic machinery was identified within the *Hormoscilla* genomes, suggesting that another member of the sponge holobiont, possibly the sponge itself, is producing the abundant sesquiterpenes often observed in *L. herbacea*. The shared NRPS cluster, with an ANI of 95.47%, has homology to shinorine, a mycosporine-like amino acid (MAA) (47). MAAs are common cyanobacterial metabolites with UV-protective properties that may also contribute to environment amelioration as compatible osmolytes and antioxidants, suggesting a benefit that the cyanobacterium may provide to its host sponge (48). The remaining common clusters are uncharacterized, including two ribosomally synthesized and posttranslationally modified peptides (RiPPs), with ANIs of 96.74% and 97.56%, and an enediyne type I polyketide synthase (T1PKS), with an ANI of 97.82%, without predictable product structures. Overall, these filamentous cyanobacteria maintain a large biosynthetic repertoire for making specialized secondary metabolites, as is common in late-branching cyanobacteria (49).

Two of the BGCs, each unique to one of the GUM_hs genomes, were subjected to further chemical characterization. The first is an expanded PBDE cluster in GUM202_hs, larger than any previously described, which contains an extra halogenase gene. GUM007 does not contain PBDEs and lacks the PBDE gene cluster (8) and yet contains a bioinformatically predictable NRPS cluster similar to the aeruginoside BGC (50).

### Genetic expansion in *hs_bmp* cluster leads to structural variety of PBDEs.

**FIG 4:**
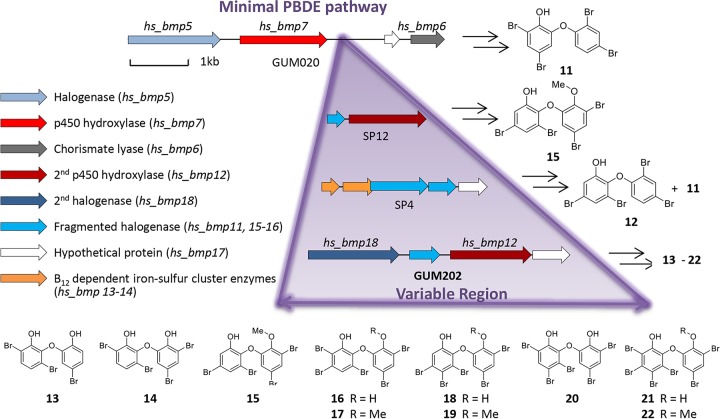
Expansion of hs_bmp in GUM202_hs leads to structurally varied PBDEs. The *hs_bmp* cluster, minimally made up of a halogenase (*hs_bmp5*), a p450 hydroxylase (*hs_bmp7*), and a chorismate lyase (*hs_bmp6*), has been shown to be responsible for the production of PBDEs. In addition to these core genes, populations of *Hormoscilla* from different clades of *Dysideidae* sponges (specimens SP12, SP4, and GUM202) contain extra genes in a variable region that correspond to the chemistry seen in each sponge.

Previous investigations of three *Hormoscilla* symbiont clades revealed that PBDE biosynthesis is encoded in the semivariable *hs_bmp* gene cluster (Fig. 4) (8). At the minimum, three core enzymes are needed to assemble PBDEs: Bmp5, a flavin-dependent brominase; Bmp6, a chorismate lyase; and Bmp7, a cytochrome P450 that couples the two brominated phenolic rings (51). Using these fundamental biosynthetic features, we queried the genomes of GUM202_hs and GUM007_hs. While the *hs_bmp* gene cluster is absent from GUM007_hs, in accordance with its secondary metabolite profile, GUM202_hs possesses an expanded *bmp* gene cluster (Fig. 4). Previously, we observed a variable genomic region between *hs_bmp6* and *hs_bmp7* in the three sequenced *hs_bmp* clusters, one of which contains hs_Bmp12, a cytochrome P450 hydroxylase that was shown to be responsible for further hydroxylated PBDEs exclusive to clade Ia sponges. The variable region in the GUM202 *hs_bmp* pathway also contains *hs_bmp12* with 99.3% pairwise identity to *hs_bmp12* from clade Ia, as well as a gene encoding a second putative flavin-dependent halogenase, *hs_bmp18*, with 78% pairwise nucleotide identity to the GUM202 *hs_bmp5*.

Based on the genomic analysis of the *hs_bmp* cluster in GUM202_hs, we predicted that PBDEs isolated from this sponge would have a higher degree of halogenation and hydroxylation than PBDEs previously characterized from metagenome-sequenced *L. herbacea* sponges (8). LC-MS analysis of methanol extracts from GUM202 revealed 10 distinct PBDEs (13–22) (Fig. S5), a majority of which are penta- and hexabrominated. We isolated the highly brominated species by preparative HPLC and characterized their structures by comparing tandem mass spectrometry and comprehensive NMR spectroscopy (Text S1) to previously reported PBDEs from *L. herbacea* specimens (8, 52). Notably, one of the aromatic rings remains unchanged as a dibromophenol moiety, while the second phenol ring varies extensively in its bromination patterns in compounds 13 to 22. We suspect that *hs_bmp18* functions as the additional brominase of the dihydroxy-PBDE 15 to give the pentabrominated 16 and 18 and the hexabrominated 21 and their respective *O*-methylated products. Compounds 13, 14, and 20 are, however, more difficult to rationalize and suggest novel debromination and/or bromine isomerization biochemistry.

10.1128/mBio.00821-19.6 FIG S5 PBDE characterization. The HPLC chromatogram (210 nm) shows the 10 PBDEs in the crude DCM:MeOH extract of GUM202. Hi-Res LC-MS/MS spectra were collected for each of the peaks identified to verify the level and distribution of bromination across the diphenyl ether scaffold. Download FIG S5, PDF file, 0.1 MB. Copyright © 2019 Schorn et al. 2019 Schorn et al. This content is distributed under the terms of the Creative Commons Attribution 4.0 International license.

### Genome mining leads to discovery of novel dysinosins.

**FIG 5:**
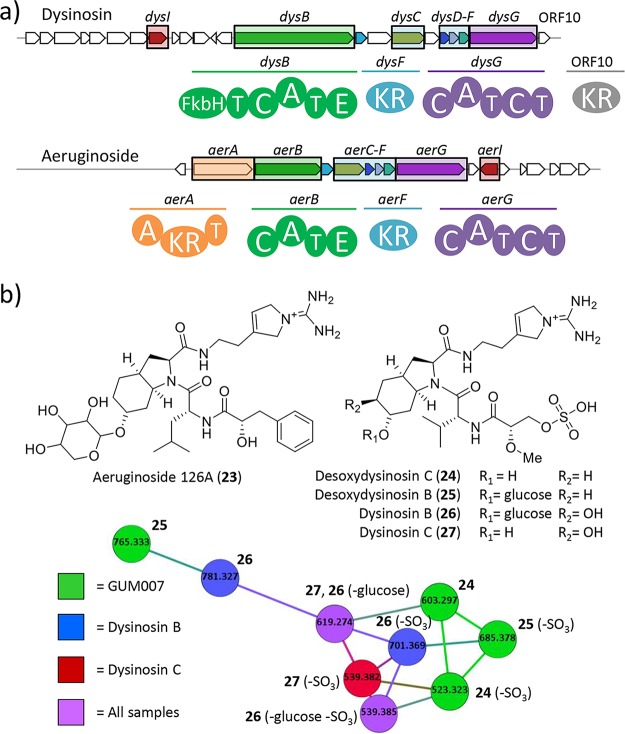
Gene cluster comparison and molecular network of dysinosins. Novel desoxydysinosin discovery through genome mining. (a) Comparison of the gene cluster found in GUM007_hs to that for aeruginoside 126A (compound 23), with homologous *aer* and *dys* genes highlighted, as well as domain structures delineated. (b) The molecular network includes standards of dysinosins B (compound 26) and C (compound 27) and two new desoxydysinosins (compounds 24 and 25) from GUM007. A small amount of compound 27 was also observed in the sample of GUM007. Domain abbreviations: FkbH, FkbH-like domain; T, thiolation domain; C, condensation domain; A, adenylation domain; E, epimerase domain; KR, ketoreductase domain.

Within the genome of GUM007_hs, but not GUM202_hs, we identified an NRPS cluster homologous to the aeruginoside BGC from the cyanobacterium Planktothrix agardhii CYA126/8 (50) (Fig. 5a). The GUM007_hs NRPS cluster contains genes homologous to *aerB*, an NRPS gene with an adenylation (A) domain selective for leucine; *aerC* to -*G*, genes responsible for the assembly and attachment of the unusual Choi amino acid moiety; and *aerI*, a putative glycosyltransferase. The only notable gene missing is *aerA*, the NRPS loading domain that installs the phenylpyruvate unit in aeruginoside (23). We thus suspected that the GUM007_hs *aer*-like BGC (named *dys*) might encode the synthesis of the structurally related dysinosins that primarily differ in their N-terminal unit, a sulfated glyceric acid. The *aerB* homologue in GUM007_hs, *dysB*, encodes a substantially larger octadomain protein with an unusual sulfotransferase domain consistent with dysinosin’s diagnostic sulfated glyceric acid. Additional adenomethyltransferase and FkbH domains in DysB are implicated in constructing the sulfated glyceric acid moiety. DysB further contains two peptidyl carrier protein (PCP) domains, one condensation (C) domain, an A domain with no consensus specificity predicted by bioinformatic tools, and an epimerization (E) domain. Taken all together, DysB is a large multimodular NRPS-type complex that appears to be responsible for the activation of a sulfated glyceric acid and addition of an unspecified d-amino acid.

The logical bioinformatic basis for a putative dysinosin cluster led us to examine GUM007 extracts for dysinosin-like molecules (18, 19). Dysinosins are potent inhibitors of the blood coagulation cascade factor VIIa and the serine protease thrombin (18). We discovered masses representing two new desoxydysinosins (compounds 24 and 25) related in structure to dysinosins B (compound 26) and C (compound 27) containing one hydroxyl group on the Choi moiety instead of two (Fig. 5b). The new dysinosins were found to have masses of 603.2806 (compound 24, 0.17-ppm error from theoretical *m/z* 603.2807) and 765.3327 (compound 25, 1.04-ppm error from theoretical *m/z* 765.3335). Interrogation of the MS/MS data revealed that in each of the dysinosin standards 26 and 27, a neutral mass loss is observed, which is consistent with the loss of sulfate from the terminal glyceric acid residue. We observed the same neutral mass loss in the new GUM007 dysinosins 24 (Fig. S6) and 25 (Fig. S7). GNPS molecular networking (53) produced a network of parent masses and corresponding neutral mass loss for both standards and the two new dysinosins (Fig. 5b). The combined MS/MS data, molecular networking, and genomic information support the discovery of two new dysinosin molecules using genome-mining-guided isolation. Understanding the biosynthetic basis of these bioactive molecules may provide a platform for rational molecule design for more effective and selective inhibitors in the blood coagulation cascade (18, 19).

10.1128/mBio.00821-19.7 FIG S6 MS/MS spectra of desoxydysinosin C (compound 24). The upper panel shows the MS^1^ of compound 24. The lower panel shows the MS^2^ for the 603.2806 parent mass, with structures of observed fragment ions. Download FIG S6, PDF file, 0.2 MB. Copyright © 2019 Schorn et al. 2019 Schorn et al. This content is distributed under the terms of the Creative Commons Attribution 4.0 International license.

10.1128/mBio.00821-19.8 FIG S7 MS/MS spectra of desoxydysinosin B (compound 25). The upper panel shows the MS^1^ of compound 25. The lower panel shows the MS^2^ for the 765.3327 parent mass, which is the glycosylated version of compound 24. Similar fragmentation is seen in the MS^2^ for compound 25; fragments labeled with an asterisk match those seen in compound 24 (Fig. S6). Download FIG S7, PDF file, 0.07 MB. Copyright © 2019 Schorn et al. 2019 Schorn et al. This content is distributed under the terms of the Creative Commons Attribution 4.0 International license.

### Conclusions.

Marine sponges continue to be one of the most promising sources of marine natural products, but challenges remain in obtaining meaningful amounts of a molecule and identifying the true producer of a given natural product. Physically vulnerable sponges are thought to rely on their microbial communities for specialized chemical defense, making the sponge microbiome a promising, untapped source of new natural products. Until recently, the full biosynthetic potential of uncultured symbionts remained concealed in their inaccessible genomes, but advances in sequencing read length and metagenomic assembly are beginning to reveal symbionts as important subjects for genome mining efforts.

The high-quality draft genomes generated for two *Hormoscilla* populations afforded a complete look at the biosynthetic capacity of these uncultivated symbionts and represent the strongest evidence yet for cyanobacterial production of multiple classes of compounds isolated from sponges, including the PBDEs and dysinosins. The variety of secondary metabolite classes encoded in the genome of just one symbiont presents a concise strategy for the sponge host to gain chemical diversity while maintaining a relationship with one dominant bacterial strain, rather than a diversified community of secondary metabolite-producing bacteria. This genomic information identified a new type of brominating enzyme that appears to directly brominate hydroxy-PBDEs and also led to the discovery of two new dysinosins and the genetic basis to begin understanding the biosynthesis of this bioactive family of compounds.

The high-quality draft genomes also allowed for reliable comparisons between *Hormoscilla* population genomes and well-characterized free-living cyanobacteria, exposing multiple avenues for exploring these symbionts’ recalcitrance to cultivation in the lab. We observed incomplete pathways of essential primary metabolites, including histidine, thiamine, and biotin. Although they are as yet unable to be cultured in the lab, these symbionts defy the strategy taken by many obligate symbionts and retain a large genome. Their expanded chemical repertoire may discourage large-scale genome streamlining. Obtaining high-quality genomes from metagenomes is the next frontier in microbiology and will begin to populate sequence databases with uncultivated strains, ultimately giving us a better look into their symbiotic lives and their yet-unseen potential for bioactive metabolite production.

## MATERIALS AND METHODS

### Hybrid sequencing and assembly of enriched *Hormoscilla* populations.

GUM007 and GUM202 were collected in July 2015 by snorkel in Guam in Pago Bay (8) and in December 2016 by scuba diving in 20 to 40 feet of water at Anae Island, respectively. Both sponges were processed to obtain enriched cyanobacterial fractions, according to a trichome enrichment protocol described previously (8). The enriched cyanobacterial fractions were subjected to genomic DNA isolation using a previously published protocol (54) to obtain high-molecular-weight DNA. Preparation of all libraries and sequencing were performed by the UC San Diego Institute for Genomic Medicine. PacBio libraries were constructed from the enriched, high-molecular-weight DNA for both GUM007 and GUM202 (see Fig. S1 in the supplemental material). PacBio sequencing libraries were generated using SMRTbell template preparation reagent kits (Pacific Biosciences), and libraries of >6 kb were selected using a PippinHT (Sage Science). Libraries were sequenced on a PacBio RS II sequencer (UCSD IGM Genomics Center, La Jolla, CA) via 4-h movies using the DNA/polymerase binding kit version P6 V2 with C_4_ sequencing chemistry. Unenriched metagenomic DNA was extracted from GUM202 whole-sponge tissue frozen in RNAlater as previously described (8). Illumina TruSeq libraries were constructed from bulk metagenomic sponge DNA for GUM202 and enriched trichome DNA for GUM007 and sequenced on an Illumina HiSeq 2500 using a 2- by 150-bp paired-end sequencing strategy.

The GUM007_hs and GUM202_hs genomes were assembled using two slightly different methods. The GUM007 genome was assembled as follows: paired-end Illumina reads were quality filtered and trimmed using Trimmomatic version 0.35 (55), with the parameters LEADING:3, TRAILING:3, HEADCROP:9, SLIDINGWINDOW:4:15, MINLEN:100, and then assembled with IDBA-UD version 1.1.1 set to default parameters (56). Preliminary contigs were grouped into bins based on percent GC, nucleotide composition, assembly depth of coverage, and taxonomic assignment by DarkHorse version 1.5 (57), as previously described (58). Reads were mapped to contigs classified as cyanobacteria using the end-to-end mode of Bowtie2, version 2.218 (59). Coverage depth was calculated using the idxstats module of SAMtools version 0.1.191 (60). Read subsets from scaffold bins identified as potentially belonging to *Cyanobacteria* were reassembled using Celera Assembler version 8.3 (61), configured with merSize = 18, utgGenomeSize = 6 Mb, and utgErrorRate = 0.02. PacBio reads were recruited to scaffolds from this assembly based on blastn E values of 1e−7 or better, filtered for a minimum size of 500 nt or greater, and then used to scaffold the Illumina assembled genome into longer sequences using SSPACE-LongRead (62).

The GUM202_hs genome was obtained by first assembling paired 150-bp Illumina reads from the GUM202 *L. herbacea* metagenome with IDBA-UD, using read trimming and assembly parameters as described above. All scaffolds were taxonomically assigned using the same strategy as described in reference 58 but employing DarkHorse version 2, which used a database customized to include the GUM007 genome. *Hormoscilla* scaffolds were binned based on shared taxonomic assignment, sequence composition, and read coverage information in Anvi’o, version 2.2.2 (63). *Hormoscilla*-associated reads were identified by mapping the Illumina data set to this bin using the local mode of Bowtie2, version 2.2.7. The PacBio reads were also phylogenetically associated with cyanobacteria by BLASTing against a custom-made database of protein-coding sequences from the GUM007_hs genome previously assembled and a database made of protein-coding sequences for all *Oscillatoria* genomes in the Joint Genome Institute (JGI) database (64). Any PacBio reads that contained a hit to one of these databases was retained. The raw, binned Illumina reads and the raw positive-hit PacBio reads were then used for assembly with hybridSPAdes in SPAdes version 3.10.1 using standard parameters (65). The Illumina reads and the GUM202_hs hybrid assembly were then used as input for GapFiller to obtain the final assembly (66).

Quality and completeness of genome assemblies were determined using CheckM version 1.0.11 using the lineage workflow (31). Average nucleotide identity (ANI) and average amino acid identity (AAI) were calculated by submitting both genome assemblies and the shared gene clusters to the ANI/AAI calculator at http://enve-omics.ce.gatech.edu/. The genome synteny plot was created using the MUMmer version 4.0.0 suite, including NUCmer for alignment, delta-filter for filtering, and mummerplot for visualization using the –fat and –large options (67). Gene cluster synteny images were created using MultiGeneBlast (68).

### Phylogenetic analysis.

A multilocus sequence analysis (MLSA) was used to build a phylogenetic tree using 25 conserved housekeeping genes (Table S2) from a set of 31 genes previously defined for bacterial MLSA (69). All 442 available cyanobacteria genomes in JGI as of 22 January 2018 were searched for the 25 housekeeping genes, of which 305 genomes contained each gene in one copy. After eliminating duplicate genomes and limiting redundant species to five representatives, a final set of 197 cyanobacterial genomes were used in the MLSA (Text S1). Chloroflexus aurantiacus J-10-fl was used as an outgroup. Each of the 25 gene sets was individually aligned using MAFFT version 7.310 (70) with high-accuracy local iterative mode using 100 iterations. Next, each alignment was trimmed using trimAl version 1.2rev59 (71) and the “automated1” option optimized for maximum-likelihood tree construction. The resulting trimmed, aligned files were concatenated using a custom Python script, and the resulting supermatrix was processed with IQ-TREE version 1.6.1 (72) with 1,000 ultrabootstrap replicates using UF:Boot2 (73) and ModelFinder (74) for each gene partition. The tree was visualized using interactive tree of life (iTOL) version 3 (75).

10.1128/mBio.00821-19.10 TABLE S2 Cyanobacterial housekeeping genes used in MLSA. Twenty-five genes were chosen to construct an MLSA of cyanobacteria. Because many of the cyanobacteria in the JGI-IMG/MER database do not have all 31 genes in single copy, the data set was narrowed to 25 conserved genes to retain more genomes in the analysis. Download Table S2, PDF file, 0.4 MB. Copyright © 2019 Schorn et al. 2019 Schorn et al. This content is distributed under the terms of the Creative Commons Attribution 4.0 International license.

### Primary metabolism and secondary metabolism analyses.

The primary metabolism analysis consisted of an initial BLAST of Synechococcus elongatus PCC 7942 protein-coding regions against both GUM_hs genomes with an E value cutoff of e−20. Missing genes were converted into their corresponding KEGG Ontology (KO) and pfam numbers for analysis in JGI IMG/MER. KOs and pfams that were absent in both *Hormoscilla* genomes were cross-referenced against gene essentiality in *S. elongatus*. The intersection of the two data sets was a set of seven genes missing in the GUM_hs genomes and essential in *S. elongatus* PCC 7942 (Table S1). In order to determine if the pathways containing missing genes were actually incomplete, ec2kegg (76) was used to generate metabolic maps for pathway comparison in *S. elongatus* PCC 7942 and the GUM_hs genomes (Fig. S3 and S4).

For secondary metabolism analysis, the two genomes were submitted to antiSMASH 4.1.0 with standard options (77). Biosynthetic gene cluster networking was done using a locally installed version of the BiG-SCAPE software with the local option enabled (46). The resulting pairwise scores were filtered for those above 0.40 and were visualized as a network using Gephi version 0.9.1 (78).

### Chemical structure elucidations.

Lyophilized GUM202 sponge tissue (1.5 g) was pulverized and extracted with 3× 20-ml methanol (MeOH) for 30 to 60 min on a benchtop nutator. The combined extracts were dried *in vacuo,* resuspended in dichloromethane (CH_2_Cl_2_), and partitioned against water. The resulting CH_2_Cl_2_ layer was dried using magnesium sulfate, filtered, and dried *in vacuo*. Preparative HPLC solvents were HPLC-grade water with 0.1% trifluoroacetic acid (TFA) and HPLC-grade acetonitrile (MeCN) with 0.1% TFA. An Agilent 218 purification system (ChemStation software; Agilent) equipped with a ProStar 410 automatic injector, Agilent ProStar UV-Vis dual-wavelength detector, 440-LC fraction collector, and an Agilent Pursuit XRs 5 C_18_ 100- by 21.2-mm preparative HPLC column was used (0 to 5 min 5% MeCN isocratic, 5 to 10 min 10 to 25% MeCN, 10 to 35 min 65 to 75% MeCN, 35 to 40 min 75 to 100% MeCN, and 40 to 45 min 100% MeCN isocratic). Ten fractions were collected and analyzed using LC-MS/MS performed on an Agilent 1260 LC system with diode array detector and a Phenomenex Kinetex 5μ C_18_(2) 100-A, 150- by 4.6-mm column in negative mode. LC-MS solvents were LC-MS-grade water with 0.1% formic acid and LC-MS-grade acetonitrile with 0.1% formic acid. Mass spectra were analyzed with Agilent MassHunter qualitative analysis version B.05.00. Purified PBDEs were validated by 1D and 2D NMR on a JEOL 500-MHz NMR spectrometer in deuterated methanol. Chemical shift trends were matched to previously reported PBDEs (52).

For the desoxydysinosins, GUM007 whole-sponge tissue was pulverized and extracted with 3× 20-ml MeOH for 30 to 60 min on a benchtop nutator. The combined extracts were dried *in vacuo*. Approximately 100 mg MeOH extract and 40 ml RNAlater solution used to store the sponges were loaded onto 1-g C_18_ solid-phase extraction columns (Canadian Life Science) and fractionated from 5% to 100% MeCN. Three standards, dysinosin A (compound 10), dysinosin B (compound 27), and dysinosin C (compound 28), kindly provided by Ron Quinn from Griffith University, were prepared as 0.1-mg/ml solutions in MeOH and analyzed with the GUN007 MeCN fractions using the Agilent 6530 Accurate-Mass Q-TOF MS mentioned above with a Phenomenex Kinetex 5 μ C_18_(2) 100-A, 150- by 4.6-mm column (0 to 3 min 5% MeCN isocratic, 3 to 23 min 5 to 100% MeCN, 23 to 26 min 100% MeCN isocratic at 0.7 ml/min). The dysinosin standards revealed a characteristic loss of sulfate, which led to the discovery of the desoxydysinosins, present in the 15% and 20% MeCN fractions. Molecular networking, using the GNPS platform (53), was used to visualize the dysinosin molecules.

### Data availability.

The GUM007 Whole-Genome Shotgun project has been deposited at DDBJ/ENA/GenBank under the accession numbers RFFB00000000, BioSample SAMN10266268, and BioProject PRJNA320446. The version described in this paper is RFFB01000000. The GUM202 Whole-Genome Shotgun project has been deposited at DDBJ/ENA/GenBank under the accession numbers RFFC00000000, BioSample SAMN10266269, and BioProject PRJNA320446. The version described in this paper is RFFC01000000.
