# Supplementary material for: Comparative Genomics of Cyanobacterial Symbionts Reveals Distinct, Specialized Metabolism in Tropical Dysideidae Sponges
Source: mBio. 2019 May 14;10(3):e00821-19. doi: 10.1128/mBio.00821-19 (PMC6520454; doi:10.1128/mBio.00821-19)
Supplement: FIG S1 [file mBio.00821-19-sf001.pdf]

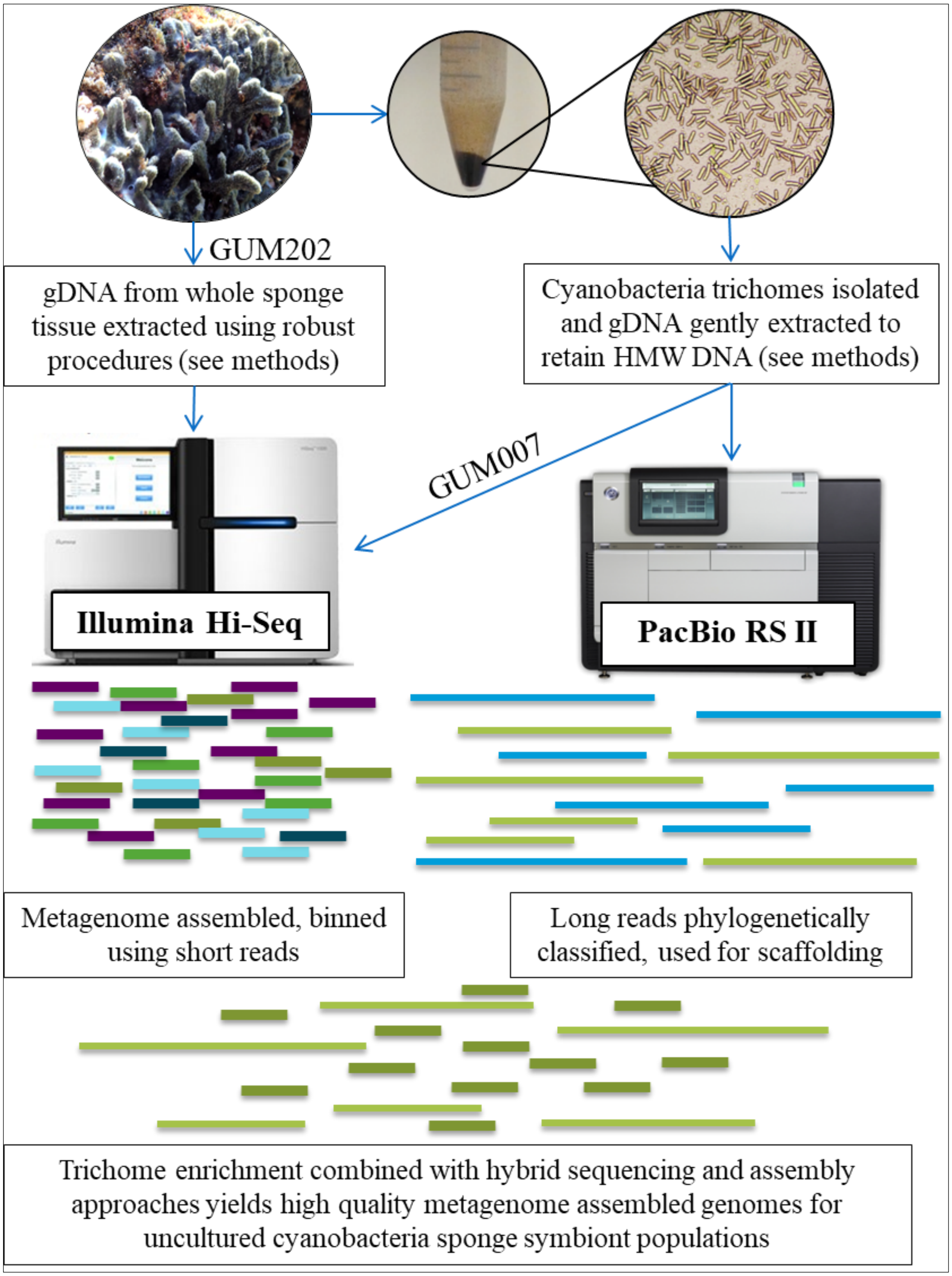

Figure S1. Workflow for processing whole sponges and enriched cyanobacteria fractions

Simplified workflow showing parallel processing and sequencing for whole sponge metagenomes using Illumina and enriched cyanobacteria using PacBio, requiring high molecular weight (HMW) DNA for long-read sequencing. Two different hybrid assembly methods were used to obtain genome assemblies.
