# Supplementary material for: Comparative Genomics of Cyanobacterial Symbionts Reveals Distinct, Specialized Metabolism in Tropical Dysideidae Sponges
Source: mBio. 2019 May 14;10(3):e00821-19. doi: 10.1128/mBio.00821-19 (PMC6520454; doi:10.1128/mBio.00821-19)
Supplement: FIG S2 [file mBio.00821-19-sf002.pdf]

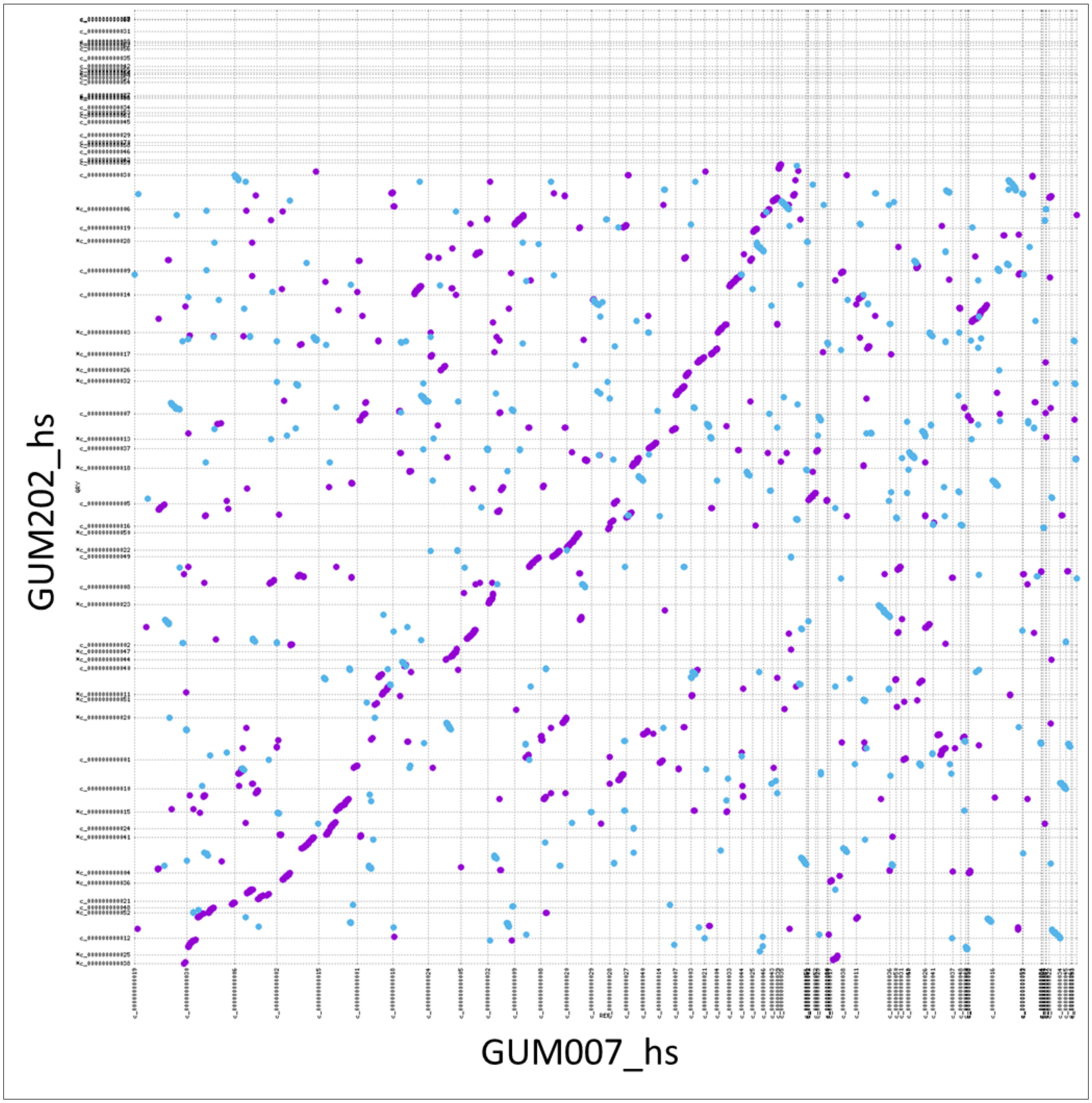

Figure S2. MUMmer plot showing synteny between GUM007_hs and GUM202_hs

Overall synteny of the two symbiont genomes, with purple dots representing forward regions of the genome that align and blue dots representing reverse regions of the genomes that align.
