# Supplementary material for: Comparative Genomics of Cyanobacterial Symbionts Reveals Distinct, Specialized Metabolism in Tropical Dysideidae Sponges
Source: mBio. 2019 May 14;10(3):e00821-19. doi: 10.1128/mBio.00821-19 (PMC6520454; doi:10.1128/mBio.00821-19)
Supplement: TABLE S1 [file mBio.00821-19-st001.pdf]

S. elongatus **PCC 7942 Locus tag**	**KO / pfam / EC**	**Name**	**Associated pathway**	**Pathway Incomplete?**
Synpcc7942_0125	K01693 / pfam00475 / 4.2.1.19	imidazoleglycerol-phosphate dehydratase (hisB)	Histidine metabolism	Yes
Synpcc7942_0475	K03689 / pfam03742	cytochrome b6-f complex subunit 8 (petN)	Photosynthesis	No; also missing in *M. producens*
Synpcc7942_0849	K08973 / pfam03653	putative membrane protein (K08973)	Chlorophyll Biosynthesis	No; HemY present
Synpcc7942_1044	K03465 / pfam02511 / 2.1.1.148	thymidylate synthase (FAD) (thyX, thy1)	Pyrimidine metabolism	No; ThyA present
Synpcc7942_1447	K01673 / pfam00484 / 4.2.1.1	carbonic anhydrase (cynT, can)	Nitrogen Metabolism	No; CcmM present
Synpcc7942_2062	K06443 / pfam05834 / 5.5.1.19	lycopene beta-cyclase (lcyB, crtL1, crtY)	Carotenoid Biosynthesis	No; CruA and CruP present
Synpcc7942_2290	K00979 / pfam02348 / 2.7.7.38	3-deoxy-manno-octulosonate cytidylyltransferase (CMP-KDO synthetase) (kdsB)	Lipopolysaccharide biosynthesis	Cannot biosynthesize Kdo
