# Supplementary material for: Comparative Genomics of Cyanobacterial Symbionts Reveals Distinct, Specialized Metabolism in Tropical Dysideidae Sponges
Source: mBio. 2019 May 14;10(3):e00821-19. doi: 10.1128/mBio.00821-19 (PMC6520454; doi:10.1128/mBio.00821-19)
Supplement: FIG S3 [file mBio.00821-19-sf003.pdf]

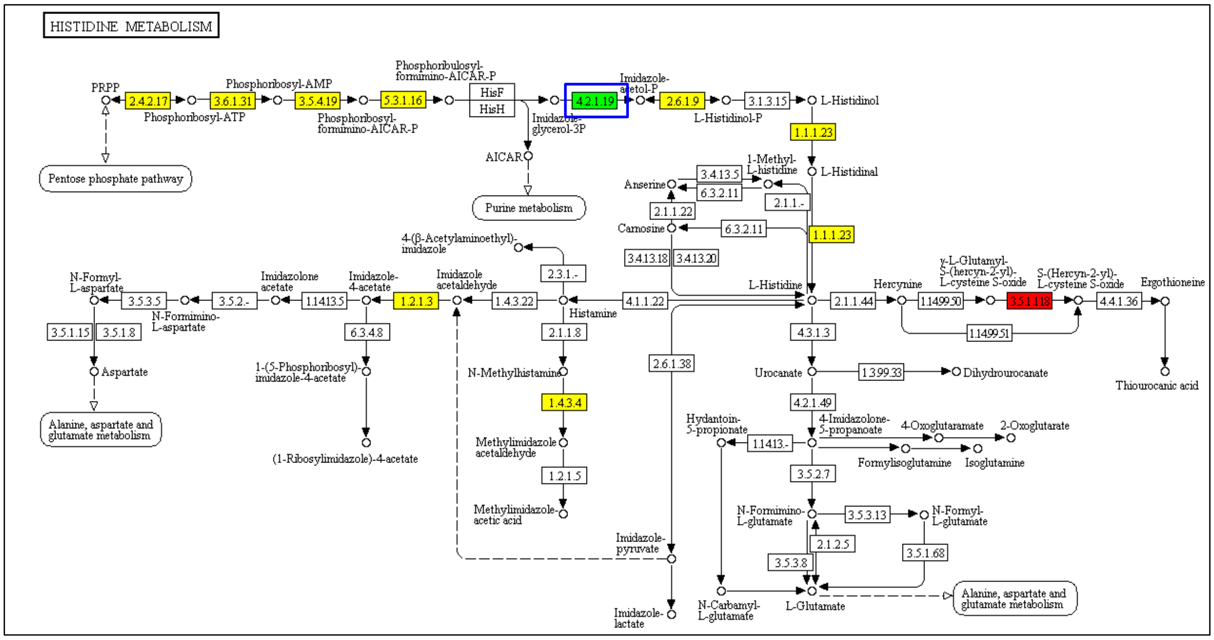

Figure S3. Histidine Metabolism in *Hormoscilla* vs *S. elongatus* PCC7942

This metabolic map was made using ec2kegg comparing histidine metabolism in *Hormoscilla* vs *S. elongatus* PCC7942. Yellow boxes indicate the corresponding EC number is present in both reference and query genomes. Green boxes are EC numbers only found in the reference genome (*S. elongatus* PCC 7942). Red boxes are EC numbers present only in the query genome (GUM007_hs and GUM202_hs). Comparison of the biosynthetic pathway for the amino acid, histidine, in S. *elongatu*s, GUM007_hs, and GUM202_hs shows an apparent lack of an essential enzyme, imidazoleglycerol-phosphate dehydratase (EC 4.2.1.19, blue box), which performs the sixth step in histidine biosynthesis.
