# Supplementary material for: Comparative Genomics of Cyanobacterial Symbionts Reveals Distinct, Specialized Metabolism in Tropical Dysideidae Sponges
Source: mBio. 2019 May 14;10(3):e00821-19. doi: 10.1128/mBio.00821-19 (PMC6520454; doi:10.1128/mBio.00821-19)
Supplement: TEXT S1 [file mBio.00821-19-s0001.pdf]

Supplementary Information Text S1

Comparative genomics of cyanobacterial symbionts reveals distinct, specialized metabolism in tropical Dysideidae sponges

Michelle A. Schorn[a], Peter A. Jordan[a], Sheila Podell[a], Jessica M. Blanton[a], Vinayak Agarwal[a,b], Jason S. Biggs[c], Eric E. Allen[a,d,e], Bradley S. Moore[a,d,f,#]

[a]Center for Marine Biotechnology and Biomedicine, Scripps Institution of Oceanography, University of California, San Diego, California, USA.

[b]School of Chemistry and Biochemistry, School of Biological Sciences, Georgia Institute of Technology, Atlanta, Georgia, USA.

[c]University of Guam Marine Laboratory, UoG Station, Guam, USA.

[d]Center for Microbiome Innovation, University of California, San Diego, California, USA.

[e]Division of Biological Sciences, University of California, San Diego, California, USA.

[f]Skaggs School of Pharmacy and Pharmaceutical Sciences, University of California, San Diego, California, USA.

Running title: Comparative genomics of cyanobacteria sponge symbionts

[#]Address correspondence to bsmoore@ucsd.edu, Tel: 858-822-6650

JGI Genome IDs used in MLSA.

Of the 442 genomes classified as cyanobacteria in the Joint Genome Institute (JGI) integrated microbial genomes and microbiomes (IMG/M) database, 305 were chosen that had 25 housekeeping genes in single copy (Table S2). Duplicate genomes were removed and overrepresented taxa were narrowed down to five representative strains. For example, there are over thirty *Prochlorococcus marinus* strains, so five were used in the final MLSA. Other abundant taxa include *Microcystis aeruginosa*, *Planktothrix* spp., and *Synechococcus* spp. The final JGI Genome ID numbers for 198 genomes used in MLSA are as follows:

641228474, 2513237397, 2503982047, 2675903261, 2561511140, 2724679208, 2506485002, 2651869734, 2630968589, 2585427799, 2507262036, 650377906, 645951858, 2687453185, 2751185742, 646311970, 2617271233, 2718217921, 2593339233, 2568526428, 2554235000, 2654587977, 2503982036, 2505679032, 2510436000, 2548877023, 2651869645, 2505679029, 2503538021, 647533184, 2582580537, 2504643013, 2531839001, 2503283007, 2623620439, 2623620440, 2503707009, 2512564012, 2528768021, 2503283023, 2508501011, 2617271122, 2757320922, 647533126, 641522622, 640612201, 643348533, 643348534, 648028021, 643348535, 647000233, 2728369583, 2509276056, 2751185667, 2516653039, 2516653040, 2517572024, 2548876996, 2548876995, 2687453106, 2516653082, 2512875027, 2516143000, 2548876998, 2509601027, 2522572068, 2523533517, 2531839741, 2523533519, 2503538020, 2509601046, 2675903523, 2671180423, 2558309063, 637000121, 2508501033, 2503754017, 2757320732, 2503538028, 2747842407, 2508501075, 2509601031, 2576861623, 2617271295, 2690315883, 2687453458, 2681813363, 2517572073, 2509601039, 2503754048, 2576861326, 639857035, 2517093042, 2509276031, 2506210028, 2505119011, 2645727866, 2731957844, 2537562159, 2534681681, 2534681685, 2645727631, 2531839541, 2630969007, 2515154003, 2506381026, 2630969006, 2718217854, 2630968268, 2597490276, 2636415546, , 2562617131, 639857037, 2744054834, 648028001, 2617270889, 2630969032, 2630968269, 2630969009, 2630968271, 2630969011, 2671180707, 2503707008, 637000199, 2509601032, 2758568003, 2556921048, 2660238729, 2503982035, , 648276706, 2510065010, 2585428050, 2627853604, 2585428053, 2636416084, 2503242000, 2523533592, 2600254967, 2602041638, 2512875029, 2602041655, 2506381002, 2507262029, 2509601013, 2509276061, 2634166547, 2606217606, 2606217687, 641228501, 640069324, 2606217319, 2606217688, 2606217560, 2645727585, 2645727806, 2617271213, 2510461040, 2600255113, 2509276045, 2504557005, 2506783054, 2504643012, 2740892533, 647000303, 2510065008, 2627853561, 2627853562, 2627853560, 2524023186, 2551306141, 2506520014, 2506520011, 2503754019, 637000308, 2517572104, 649990022, 2579778681, 2509276030, 647533236, 2506520048, 2508501041, 641522654, 2504643000, 2504643002, 2718218441, 2507262052, 637000314, 2568526149, 637000315, 2517572074, 637000320, 2597489959, 2648501203, 2627853943, 646564504, 2529292565, 2508501034. 641008485 was used as an outgroup.

<u>Missing Essential Gene Analysis Supplementary Information</u>

A small subunit of the cytochrome b6-f complex, PetN, appears to be missing in both GUM_hs genomes. While PetN was determined to be essential in *S. elongatus* PCC7942, *Synechocystis* sp. PCC 6803, and *Nostoc* sp. PCC 7120, it does not appear to be essential for *M. producens*, as this reference genome also lacks PetN (1, 2). The four large subunits of the cytochrome b6-f complex, PetA, PetB, PetC, and PetD, are present in both *Hormoscilla* genomes and *M. producens*. Although their exact function in cyanobacteria remains largely unknown, three small subunits of the cytochrome b6-f complex, PetG, PetL, and PetN, have been implicated in the assembly and stability of the whole complex in plants (3). As the cytochrome b6-f complex plays an essential role in electron transfer between photosystem II and photosystem I, it is not likely that this enzyme is dysfunctional in *M. producens*, but rather that *M. producens* does not require PetN for its cytochrome b6-f complex to function (4). We therefore conclude that this missing gene does not indicate that the cytochrome b6-f complex is missing or dysfunctional in *Hormoscilla*, but that *Hormoscilla* and the related *M. producens* may not need PetN for a functioning cytochrome b60-f complex, or it may be replaced by a yet unknown protein.

We found alternative pathways in *Hormoscilla* for four other missing essential genes. The gene encoding thymidylate synthase, *thyx* (EC 2.1.1.148), is missing in both GUM_hs genomes, but upon further interrogation of the pyrimidine metabolism map, an alternative gene, *thyA* (EC 2.1.1.45), can perform the same transformation and is present in both genomes. Upon investigation of the missing essential lycopene beta-cyclase gene, *lcyB* (EC 5.5.1.19), we found that *M. producens* is also missing this gene. *M. producens* and both *Hormoscilla* genomes possess two other genes that encode lycopene cyclases, CruA and CruP, that can perform the same function of LcyB (5). The missing putative membrane protein (K08973), as annotated in *S. elongatus*, is annotated as protoporphyrinogen oxidase HemJ in *M. producens*, and has been shown to be an enzyme that replaces HemG or HemY in heme biosynthesis (6). The *Hormoscilla* genomes possess HemY (EC 1.3.3.4) to catalyze the formation of protoporphyrin IX, and thus do not require HemJ as *S. elongatus* and *M. producens* do.

Finally, the missing gene, *cynT,* encodes the enzyme carbonic anhydrase, which acts as a key player in the carbon dioxide concentrating mechanism of cyanobacteria. This type of β-carbonic anhydrase resides in the carboxysome, where in converts bicarbonate to carbon dioxide for use in photosynthesis (7). Once genomes of β-cyanobacteria began to be sequenced, it was noticed that they lacked a cytosolic β-carbonic anhydrase, but contained a CcmM protein with domains homologous to γ-carbonic anhydrases (8). The elucidation of the structure of a cyanobacterial CcmM and subsequent experiments confirmed that it acts as a carbonic anhydrase in cyanobacteria lacking a conventional β-carbonic anhydrase (9). Both *Hormoscilla* genomes contain CcmM, suggesting that it plays the role of the essential β-carbonic anhydrase in *S. elongatus*.

The essential missing gene analysis identified the *kdsB* gene (EC 2.7.7.38) involved in lipopolysaccharide biosynthesis, and indeed the entire Kds pathway, as missing in *Hormoscilla*. The Kds pathway, comprised of a suite of four enzymes, KdsA-D, is responsible for turning D-ribulose-5P into CMP-3-deoxy-D-manno-octulosonate (ketodeoxyoctonate or Kdo). *M. producens* also has the genes *kdsA-C*, and the pathway is largely conserved between plants and bacteria (10). While Kdo is often present in the sugar component of lipopolysaccharides in Gram-negative bacteria, there have been multiple cyanobacteria found to not contain Kdo (11). It is likely that *Hormoscilla* have different sugar variants in its lipopolysaccharide structure and therefore does not require Kdo.

Characterization of PBDEs from GUM202

Hi-Res LCMSMS spectra were collected for each of the peaks identified in Figure S5 to verify the level and distribution of bromination across the diphenyl ether scaffold. Compounds **13-22** were characterized by 1D and 2D NMR, guided by the strategy used in (12). Compound **14** was unable to be separated from another PBDE species, and the structure assignment was deduced from the mixture.

Compound 13: ^{1}H-NMR (500 MHz, CD_3OD) δ 7.34 (d, J = 8.7 Hz, 1H), 7.08 (d, J = 8.7 Hz, 1H), 6.99 (dd, J = 8.6, 2.3 Hz, 1H), 6.81 (d, J = 8.5 Hz, 1H), 6.37 (d, J = 2.3 Hz, 1H). **Compound 14:** ^{1}H-NMR (500 MHz, CD_3OD) 7.36 (d, J = 8.7 Hz, 1H), 7.29 (d, J = 2.2 Hz, 1H), 7.09 (d, J = 8.7 Hz, 1H), 6.35 (d, J = 2.2 Hz, 1H). **Compound 15:** ^{1}H-NMR (500 MHz, CD_3OD) δ 7.40 (d, J = 2.2 Hz, 1H), 7.36 (d, J = 2.2 Hz, 1H), 7.14 (d, J = 2.2 Hz, 1H), 6.50 (d, J = 2.2 Hz, 1H), 3.99 (s, 3H). **Compound 16:** ^{1}H-NMR (500 MHz, CD_3OD) δ 7.58 (s, 1H), 7.34 (d, J = 2.2 Hz, 1H), 6.43 (d, J = 2.2 Hz, 1H). **Compound 17:** ^{1}H-NMR (500 MHz, CD_3OD) δ 7.61 (s, 1H), 7.44 (d, J = 2.2 Hz, 1H), 6.52 (d, J = 2.2 Hz, 1H), 4.00 (s, 3H). **Compound 18:** ^{1}H-NMR (500 MHz, CD_3OD) δ 7.39 (s, 1H), 7.31 (d, J = 2.2 Hz, 1H), 6.43 (d, J = 2.2 Hz, 1H). **Compound 19:** ^{1}H-NMR (500 MHz, CD_3OD) δ 7.42 (d, J = 2.2 Hz, 1H), 7.39 (s, 1H), 6.51 (d, J = 2.2 Hz, 1H), 3.98 (s, 3H). **Compound 20:** ^{1}H-NMR (500 MHz, CD_3OD) δ 7.84 (s, 1H), 7.34 (d, J = 2.2 Hz, 1H), 6.40 (d, J = 2.2 Hz, 1H). **Compound 21:** ^{1}H-NMR (500 MHz, CD_3OD) δ 7.34 (d, J = 2.2 Hz, 1H), 6.46 (d, J = 2.2 Hz, 1H). **Compound 22:** ^{1}H-NMR (500 MHz, CD_3OD) δ 7.46 (d, J = 2.2 Hz, 1H), 6.54 (d, J = 2.2 Hz, 1H), 4.00 (s, 3H).

Fragmentation of Novel Desoxydysinosins

The in source and collision induced loss of sulfate can be seen in MS^1 and MS^2 for both desoxydysinosins, and is the most abundant species in both cases (Figs. S6 and S7). The next major fragment in **24** represents the de-sulfated core structure with a loss of the two terminal amino groups on the guanadyl moiety (m/z 481.2934). The next major fragments observed are the Choi

and guanadyl core with successive loss of terminal amino groups (m/z 322.2230, 305.1908, 280.2031). This successive loss is seen again in just the guanadyl fragment (m/z 155.1001, 140.1049, 113.1090). The 'a ion' produced when the valine is fragmented from the Choi can also be seen (m/z 174.1119), as can the 'b ion' (m/z 202.1006). Finally, the valine immonium ion is seen (m/z 72.0831). Similar fragmentation is seen in the MS^2 for **26**. Again, the main ion is the loss of sulfate (m/z 685.3779), followed by a loss of glucose (m/z 523.3259). The glycosylated fragment with a loss of sulfate and loss of both terminal amino groups from the guanadyl moiety can be seen (m/z 643.3323). The glycosylated Choi and guanadyl core is seen (m/z 484.2889) as well as the loss of both terminal amino groups (m/z 442.2736). The remaining fragments labelled with an asterisk match those seen in **24**.

1. Schneider D, Volkmer T, Rogner M. 2007. PetG and PetN, but not PetL, are essential subunits of the cytochrome b6f complex from Synechocystis PCC 6803. Res Microbiol 158:45-50.
2. Baniulis D, Yamashita E, Whitelegge JP, Zatsman AI, Hendrich MP, Hasan SS, Ryan CM, Cramer WA. 2009. Structure-Function, Stability, and Chemical Modification of the Cyanobacterial Cytochrome b6f Complex from Nostoc sp. PCC 7120*. J Biol Chem 284:9861-9.
3. Schwenkert S, Legen J, Takami T, Shikanai T, Herrmann RG, Meurer J. 2007. Role of the low-molecular-weight subunits PetL, PetG, and PetN in assembly, stability, and dimerization of the cytochrome b6f complex in tobacco. Plant Physiol 144:1924-35.
4. Baniulis D, Zhang H, Zakharova T, Hasan SS, Cramer WA. 2011. Purification and crystallization of the cyanobacterial cytochrome b6f complex. Methods Mol Biol 684:65-77.
5. Maresca JA, Graham JE, Wu M, Eisen JA, Bryant DA. 2007. Identification of a fourth family of lycopene cyclases in photosynthetic bacteria. Proc Natl Acad Sci U S A 104:11784-9.
6. Kato K, Tanaka R, Sano S, Tanaka A, Hosaka H. 2010. Identification of a gene essential for protoporphyrinogen IX oxidase activity in the cyanobacterium Synechocystis sp. PCC6803. Proc Natl Acad Sci U S A 107:16649-54.
7. Badger M. 2003. The roles of carbonic anhydrases in photosynthetic CO2 concentrating mechanisms | SpringerLink. Photosynthesis Research 77.
8. Badger MR, Price GD. 2003. CO2 concentrating mechanisms in cyanobacteria: molecular components, their diversity and evolution. J Exp Bot 54:609-22.
9. Pena KL, Castel SE, de Araujo C, Espie GS, Kimber MS. 2010. Structural basis of the oxidative activation of the carboxysomal gamma-carbonic anhydrase, CcmM. Proc Natl Acad Sci U S A 107:2455-60.
10. Smyth KM, Marchant A. 2013. Conservation of the 2-keto-3-deoxymanno-octulosonic acid (Kdo) biosynthesis pathway between plants and bacteria. Carbohydr Res 380:70-5.
11. Durai P, Batool M, Choi S. 2015. Structure and Effects of Cyanobacterial Lipopolysaccharides. Mar Drugs 13:4217-30.
12. Calcul L, Chow R, Oliver AG, Tenney K, White KN, Wood AW, Fiorilla C, Crews P. 2009. NMR strategy for unraveling structures of bioactive sponge-derived oxy-polyhalogenated diphenyl ethers. J Nat Prod 72:443-9.
