# Supplementary material for: Comparative Genomics of Cyanobacterial Symbionts Reveals Distinct, Specialized Metabolism in Tropical Dysideidae Sponges
Source: mBio. 2019 May 14;10(3):e00821-19. doi: 10.1128/mBio.00821-19 (PMC6520454; doi:10.1128/mBio.00821-19)
Supplement: FIG S4 [file mBio.00821-19-sf004.pdf]

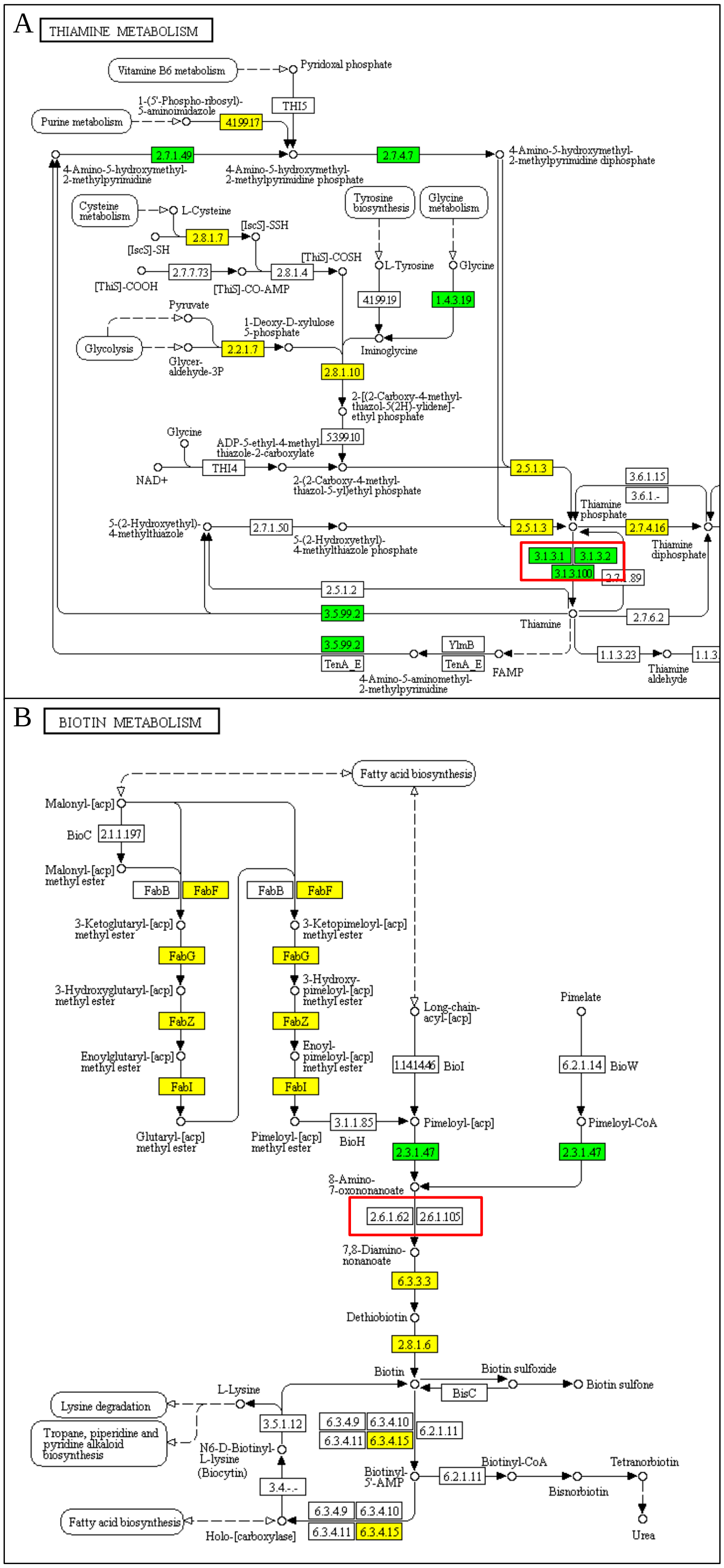
A
THIAMINE METABOLISM
Vitamine B6 metabolism
Pyridoxal phosphate
THI5
Purine metabolism
1-(5'-Phospho-ribosyl)-
5-aminoimidazole
4.1.99.17
2.7.1.49
2.7.4.7
4-Amino-5-hydroxymethyl-
2-methylpyrimidine
4-Amino-5-hydroxymethyl-
2-methylpyrimidine phosphate
4-Amino-5-hydroxymethyl-
2-methylpyrimidine diphosphate
Cysteine
metabolism
L-Cysteine
2.8.1.7
[IscS]-SSH
[IscS]-SH
Tyrosine
biosynthesis
Glycine
metabolism
[ThiS]-COSH
2.7.7.73
2.8.1.4
[ThiS]-COOH
[ThiS]-CO-AMP
L-Tyrosine
Glycine
4.1.99.19
1.4.3.19
Pyruvate
1-Deoxy-D-xylulose
5-phosphate
Glycolysis
2.2.1.7
Iminoglycine
Glycer-
aldehyde-3P
2.8.1.10
2-[(2-Carboxy-4-methyl-
thiazol-5(2H)-ylidene]-
ethyl phosphate
Glycine
ADP-5-ethyl-4-methyl-
thiazole-2-carboxylate
5.3.99.10
NAD+
THI4
2-(2-Carboxy-4-methyl-
thiazol-5-yl)ethyl phosphate
2.5.1.3
3.6.1.15
3.6.1.-
Thiamine
phosphate
5-(2-Hydroxyethyl)-
4-methylthiazole
2.7.1.50
5-(2-Hydroxyethyl)-
4-methylthiazole phosphate
2.5.1.3
2.7.4.16
Thiamine
diphosphate
3.1.3.1
3.1.3.2
3.1.3.100
2.5.1.2
3.5.99.2
Thiamine
2.7.6.2
3.5.99.2
YlmB
TenA_E
TenA_E
FAMP
1.1.3.23
Thiamine
aldehyde
4-Amino-5-aminomethyl-
2-methylpyrimidine
B
BIOTIN METABOLISM
Fatty acid biosynthesis
Malonyl-[acp]
BioC
2.1.1.197
Malonyl-[acp]
methyl ester
FabB
FabF
FabB
FabF
3-Ketoglutaryl-[acp]
methyl ester
3-Ketopimeloyl-[acp]
methyl ester
FabG
FabG
3-Hydroxyglutaryl-[acp]
methyl ester
3-Hydroxy-
pimeloyl-[acp]
methyl ester
FabZ
FabZ
Pimelate
Long-chain-
acyl-[acp]
Enoylglutaryl-[acp]
methyl ester
Enoyl-
pimeloyl-[acp]
methyl ester
1.14.14.46
BioI
6.2.1.14
BioW
FabI
FabI
3.1.1.85
Pimeloyl-[acp]
Pimeloyl-CoA
Glutaryl-[acp]
methyl ester
Pimeloyl-[acp]
methyl ester
BioH
2.3.1.47
2.3.1.47
8-Amino-
7-oxononanoate
2.6.1.62
2.6.1.105
7,8-Diamino-
nonanoate
6.3.3.3
Dethiobiotin
2.8.1.6
Biotin
Biotin sulfoxide
Biotin sulfone
BisC
L-Lysine
Lysine degradation
3.5.1.12
6.3.4.9
6.3.4.10
6.3.4.11
6.3.4.15
6.2.1.11
Tropane, piperidine and
pyridine alkaloid
biosynthesis
N6-D-Biotinyl-
L-lysine
(Biocytin)
Biotinyl-
5'-AMP
Biotinyl-CoA
6.2.1.11
Tetranorbiotin
Bisnorbiotin
3.4.-.-
6.3.4.9
6.3.4.10
6.3.4.11
6.3.4.15
Fatty acid biosynthesis
Holo-[carboxylase]
Urea

Figure S4. Thiamine and Biotin Metabolism in *Hormoscilla* vs *S. elongatus and Moorea producens*

These metabolic maps were made using ec2kegg and follow the same coloring scheme: Yellow boxes indicate the corresponding EC number is present in both reference and query genomes. Green boxes are EC numbers only found in the reference genome (*S. elongatus* PCC 7942 in S4a and *M. producens* in S4b). Figure S4a: Both GUM007_hs and GUM202_hs genomes appear to be lacking the three enzymes involved in the last step of thiamine biosynthesis (ECs 3.1.3.1, 3.1.3.2, and 3.1.3.100, red box). Figure S4b: The biotin pathway appears incomplete in both *M. producens* and *Hormoscilla*. Putative homologues of BioK, which can replace BioH in pimeloyl-CoA biosynthesis, were found in *M. producens* and *Hormoscilla*. However, no homologues of BioA (ECs 2.6.1.62 and 2.6.1.105, red box) have been found in either species.
