# Supplementary material for: Comparative Genomics of Cyanobacterial Symbionts Reveals Distinct, Specialized Metabolism in Tropical Dysideidae Sponges
Source: mBio. 2019 May 14;10(3):e00821-19. doi: 10.1128/mBio.00821-19 (PMC6520454; doi:10.1128/mBio.00821-19)
Supplement: FIG S5 [file mBio.00821-19-sf005.pdf]

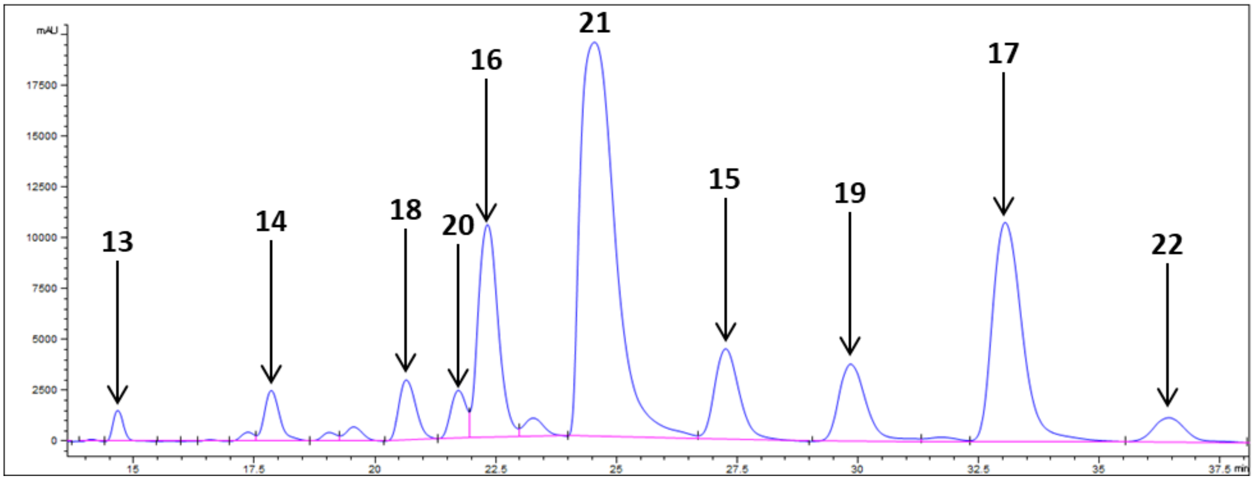

Figure S5. This HPLC chromatogram (210nm) shows the ten PBDEs in the crude DCM:MeOH extract of GUM202. Hi-Res LCMSMS spectra were collected for each of the peaks identified to verify the level and distribution of bromination across the diphenyl ether scaffold. Compounds **13-22** were characterized by 1D and 2D NMR. Compound **14** was unable to be separated from another PBDE species, and the structure assignment was deduced from the mixture. **Compound 13:** ^{1}H-NMR (500 MHz, CD_3OD) δ 7.34 (d, J = 8.7 Hz, 1H), 7.08 (d, J = 8.7 Hz, 1H), 6.99 (dd, J = 8.6, 2.3 Hz, 1H), 6.81 (d, J = 8.5 Hz, 1H), 6.37 (d, J = 2.3 Hz, 1H). **Compound 14:** ^{1}H-NMR (500 MHz, CD_3OD) 7.36 (d, J = 8.7 Hz, 1H), 7.29 (d, J = 2.2 Hz, 1H), 7.09 (d, J = 8.7 Hz, 1H), 6.35 (d, J = 2.2 Hz, 1H). **Compound 15:** ^{1}H-NMR (500 MHz, CD_3OD) δ 7.40 (d, J = 2.2 Hz, 1H), 7.36 (d, J = 2.2 Hz, 1H), 7.14 (d, J = 2.2 Hz, 1H), 6.50 (d, J = 2.2 Hz, 1H), 3.99 (s, 3H). **Compound 16:** ^{1}H-NMR (500 MHz, CD_3OD) δ 7.58 (s, 1H), 7.34 (d, J = 2.2 Hz, 1H), 6.43 (d, J = 2.2 Hz, 1H). **Compound 17:** ^{1}H-NMR (500 MHz, CD_3OD) δ 7.61 (s, 1H), 7.44 (d, J = 2.2 Hz, 1H), 6.52 (d, J = 2.2 Hz, 1H), 4.00 (s, 3H). **Compound 18:** ^{1}H-NMR (500 MHz, CD_3OD) δ 7.39 (s, 1H), 7.31 (d, J = 2.2 Hz, 1H), 6.43 (d, J = 2.2 Hz, 1H). **Compound 19:** ^{1}H-NMR (500 MHz, CD_3OD) δ 7.42 (d, J = 2.2 Hz, 1H), 7.39 (s, 1H), 6.51 (d, J = 2.2 Hz, 1H), 3.98 (s, 3H). **Compound 20:** ^{1}H-NMR (500 MHz, CD_3OD) δ 7.84 (s, 1H), 7.34 (d, J = 2.2 Hz, 1H), 6.40 (d, J = 2.2 Hz, 1H). **Compound 21:** ^{1}H-NMR (500 MHz, CD_3OD) δ 7.34 (d, J = 2.2 Hz, 1H), 6.46 (d, J = 2.2 Hz, 1H). **Compound 22:** ^{1}H-NMR (500 MHz, CD_3OD) δ 7.46 (d, J = 2.2 Hz, 1H), 6.54 (d, J = 2.2 Hz, 1H), 4.00 (s, 3H).
