# Supplementary material for: Comparative Genomics of Cyanobacterial Symbionts Reveals Distinct, Specialized Metabolism in Tropical Dysideidae Sponges
Source: mBio. 2019 May 14;10(3):e00821-19. doi: 10.1128/mBio.00821-19 (PMC6520454; doi:10.1128/mBio.00821-19)
Supplement: FIG S6 [file mBio.00821-19-sf006.pdf]

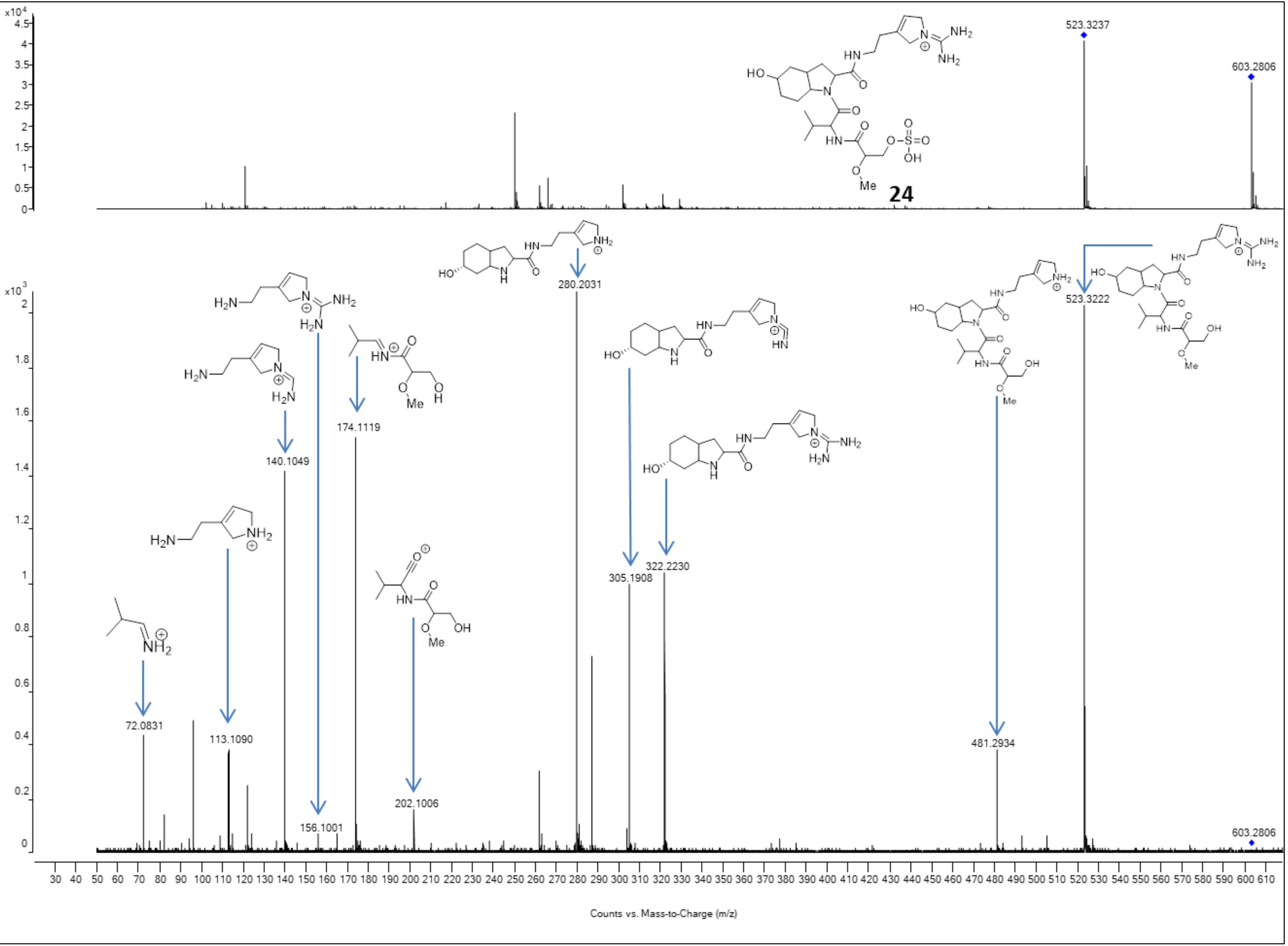

x10^4
523.3237
603.2806
24
x10^3
72.0831
113.1090
140.1049
156.1001
174.1119
202.1006
280.2031
305.1908
322.2230
481.2934
523.3222
603.2806
Counts vs. Mass-to-Charge (m/z)

Figure S6. MS/MS spectra of desoxydysinosin C (24). The upper panel shows the MS^1 of (**24**). The lower panel shows the MS^2 for the 603.2806 parent mass, with structures of observed fragment ions. The desulfated mass was always observed as both a neutral mass loss in source and via collision induced dissociation. The next major fragment in **24** represents the de-sulfated core structure with a loss of the two terminal amino groups on the guanadyl moiety (m/z 481.2934). The next major fragments observed are the Choi and guanadyl core with successive loss of terminal amino groups (m/z 322.2230, 305.1908, 280.2031). This successive loss is seen again in just the guanadyl fragment (m/z 155.1001, 140.1049, 113.1090). The 'a ion' produced when the valine is fragmented from the Choi can also be seen (m/z 174.1119), as can the 'b ion' (m/z 202.1006). Finally, the valine immonium ion is seen (m/z 72.0831).
