# Supplementary material for: Comparative Genomics of Cyanobacterial Symbionts Reveals Distinct, Specialized Metabolism in Tropical Dysideidae Sponges
Source: mBio. 2019 May 14;10(3):e00821-19. doi: 10.1128/mBio.00821-19 (PMC6520454; doi:10.1128/mBio.00821-19)
Supplement: FIG S7 [file mBio.00821-19-sf007.pdf]

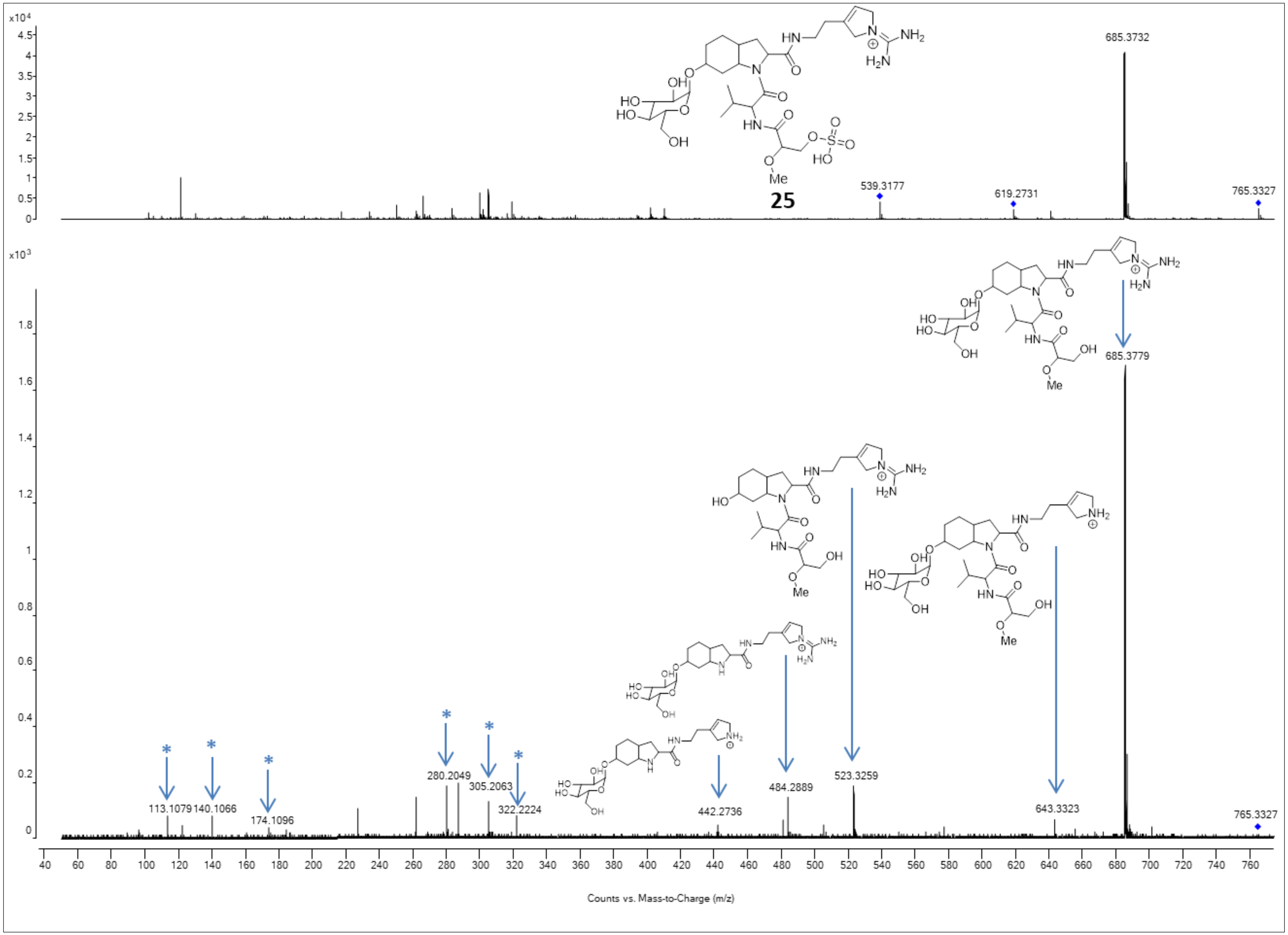

x10^4
685.3732
539.3177
619.2731
765.3327
25
x10^3
685.3779
523.3259
643.3323
484.2889
442.2736
280.2049
305.2063
322.2224
113.1079
140.1066
174.1096
765.3327
Counts vs. Mass-to-Charge (m/z)

Figure S7. MS/MS spectra of desoxydysinosin B (25) . The upper panel shows the MS^1 of **25**. The lower panel shows the MS^2 for the 765.3327 parent mass, which is the glycosylated verson of **24**. Similar fragmentation is seen in the MS^2 for **25**. Again, the main ion is the loss of sulfate (m/z 685.3779), followed by a loss of glucose (m/z 523.3259). The glycosylated fragment with a loss of sulfate and loss of both terminal amino groups from the guanadyl moiety can be seen (m/z 643.3323). The glycosylated Choi and guanadyl core is seen (m/z 484.2889) as well as the loss of both terminal amino groups (m/z 442.2736). The remaining fragments labelled with an asterisk match those seen in **24**.
