# Supplementary material for: Comparative Genomics of Cyanobacterial Symbionts Reveals Distinct, Specialized Metabolism in Tropical Dysideidae Sponges
Source: mBio. 2019 May 14;10(3):e00821-19. doi: 10.1128/mBio.00821-19 (PMC6520454; doi:10.1128/mBio.00821-19)
Supplement: TABLE S2 [file mBio.00821-19-st002.pdf]

**Gene Name**	**Abbreviation**	**pfam or TIGRFAM**
Phosphoglycerate kinase	pgk	pfam00162
Ribosomal protein L5	rplE	pfam00281
Ribosomal protein L3	rplC	pfam00297
Ribosomal protein L6	rplF	pfam00347
Ribosomal protein S10/S16	rpsI	pfam00380
Ribosomal protein S11	rpsK	pfam00411
Ribosomal protein S13/S18	rpsM	pfam00416
SsrA-binding protein	smpB	TIGR00086
translation elongation factor Ts	tsf	TIGR00116
ribosome recycling factor	frr	TIGR00496
ribosomal protein S2, bacterial type	rpsB	TIGR01011
ribosomal protein S6, bacterial/organelle type	rpsE	TIGR01021
ribosomal protein L19, bacterial type	rplS	TIGR01024
ribosomal protein L20	rplT	TIGR01032
ribosomal protein S11, bacterial/organelle	rpsJ	TIGR01049
ribosomal protein S19, bacterial/organelle	rpsS	TIGR01050
ribosomal protein L13, bacterial type	rplM	TIGR01066
ribosomal protein L14, bacterial/organelle	rplN	TIGR01067
ribosomal protein L16, bacterial/organelle	rplP	TIGR01164
ribosomal protein L1, bacterial/chloroplast	rplA	TIGR01169
ribosomal protein L2, bacterial/organellar	rplB	TIGR01171
50S ribosomal protein uL11, bacterial form	rplK	TIGR01632
transcription termination factor NusA	nusA	TIGR01953
DNA-directed RNA polymerase, beta subunit	rpoB	TIGR02013
50S ribosomal protein L4, bacterial/organelle	rplD	TIGR03953
